# Lithiated Prussian blue analogues as positive electrode active materials for stable non-aqueous lithium-ion batteries

**DOI:** 10.1038/s41467-022-35376-1

**Published:** 2022-12-16

**Authors:** Ziheng Zhang, Maxim Avdeev, Huaican Chen, Wen Yin, Wang Hay Kan, Guang He

**Affiliations:** 1 Tianjin Key Laboratory of Advanced Functional Porous Materials, Institute for New Energy Materials and Low-Carbon Technologies, School of Materials Science and Engineering, Tianjin University of Technology, Tianjin, 300384 China; 2 Renewable Energy Conversion and Storage Center (RECAST), Haihe Laboratory of Sustainable Chemical Transformations, Key Laboratory of Advanced Energy Materials Chemistry (Ministry of Education), College of Chemistry, Nankai University, Tianjin, 300071 China; 3 Australian Nuclear Science and Technology Organization (ANSTO), Lucas Heights, NSW 2234 Australia; 4 Spallation Neutron Source Science Center, Dalang, Dongguan 523803 China; 5 Institute of High Energy Physics, Chinese Academy of Sciences, Beijing, 100049 China; 6 Tianneng Co. Ltd, Huzhou, 313100 China

**Keywords:** Batteries, Design, synthesis and processing, Chemical synthesis, Materials for energy and catalysis, Energy storage

## Abstract

Prussian blue analogues (PBAs) are appealing active materials for post-lithium electrochemical energy storage. However, PBAs are not generally suitable for non-aqueous Li-ion storage due to their instability upon prolonged cycling. Herein, we assess the feasibility of PBAs with various lithium content for non-aqueous Li-ion storage. We determine the crystal structure of the lithiated PBAs via neutron powder diffraction measurements and investigate the influence of water on structural stability and Li-ion migration through operando X-ray diffraction measurements and bond valence simulations. Furthermore, we demonstrate that a positive electrode containing Li_2-x_FeFe(CN)_6_⋅nH_2_O (0 ≤ x ≤ 2) active material coupled with a Li metal electrode and a LiPF_6_-containing organic-based electrolyte in coin cell configuration delivers an initial discharge capacity of 142 mAh g^−1^ at 19 mA g^−1^ and a discharge capacity retention of 80.7% after 1000 cycles at 1.9 A g^−1^. By replacing the lithium metal with a graphite-based negative electrode, we also report a coin cell capable of cycling for more than 370 cycles at 190 mA g^−1^ with a stable discharge capacity of about 105 mAh g^−1^ and a discharge capacity retention of 98% at 25 °C.

## Abstract (web summary)

Prussian blue analogues (PBAs) are appealing materials for aqueous Na- and K- ion batteries but are limited for non-aqueous Li-ion storage. Here, the authors report the synthesis of various lithiated PBAs and discuss critical factors for improving the non-aqueous electrochemical storage of Li ions.

## Introduction

Non-aqueous lithium-ion batteries (LIBs) have become a dominant power source for portal electronic devices, power tools, electric vehicles, and other renewable energy storage systems^1^. Albeit its popularity, the thermal runaway induced accidents are occasionally happened all over the world^2,3^. In commercialized lithium-ion batteries, the layered transition-metal (TM) oxides, represented by a general formula of LiMO_2_, have been widely used as higher energy density positive electrode materials due to their appealing electrochemical performance namely the specific gravimetric capacity, rate capacity and energy density^4,5^. However, the inadequate thermal stability of the cell at charge states seems inevitable for such layered structures due to their O *2p* and M *3d* orbital overlapping. Consequently, prolonged cycling might induce undesired structural defects of cationic migration and oxide-ion vacancy. Alternatively, polyanion-type cathodes such as LiFePO_4_ demonstrate greatly promoted thermal stability due to the strong covalent P–O bonds in the structures^6–8^. Furthermore, the inductive effect (an electronic effect due to the polarisation of σ bonds within a molecule or ion) brings a higher working potential in polyanion cathodes in comparison to layered lithium metal oxide cathodes. Nonetheless most polyanion cathodes suffer from lower theoretical capacities as compared to their layered counterparts. It’s been a fundamental task to explore new cathode materials for the development of LIBs.

Prussian blue analogues (PBAs) have open channel structure that is suitable for alkali ion de/intercalation, and in certain circumstance two-electron reaction per formula unit could occur with optimized compositions^9–15^. In the past few years, intercalation behaviors of different metal ions have been studied including Li^+^, Na^+^, K^+^, Rb^+^, NH^4+^, Mg^2+^, Zn^2+^ etc., among which Na-PBA is considered the most promising applications in Na-ion batteries (SIBs) with balanced capacities, cell voltages, rate capability and cycling life^16–19^. For example, the Prussian white Na_2_FeFe(CN)_6_ has a theoretical capacity of 170 mAh g^−1^ and average cell discharge voltage of ~3 V vs. Na/Na^+^, both of which are competitive among various Na cathodes^20^. Practically, high discharge capacity (>150 mAh g^−1^), stable cycling life (>1000 cycles) and good rate capability (>1.9 A g^−1^) have been demonstrated by research groups in Na-FePBA cells^21,22^.

Despite the great of PBA materials in SIBs, no commercial SIBs are commercially available yet. On the other hand, the applications of PBA in LIBs are not optimistic due to the following concerns. First, Li intercalation potential is generally higher than for Na in most polyanion frameworks, *i.e*., FePO_4_ and VOPO_4_, but this advantage is significantly diminished for Li-PBA cathodes. Furthermore, the replacement of Na-ions by Li-ions could cause structural decomposition of PBAs. Goodenough et al. found the shorter Pauli repulsion radius of Li-ion makes it prefer to stay closer to N as compared to Na in the MnFe(CN)_6_ host, which leads to structural evolution of MnN_6_ octahedra into LiN_4_ and MnN_4_ tetrahedra^23^. Ling et al. also demonstrate that the stable interstitial site will convert from face-centered site to body-centered site with the ion radius increases^24^. All these findings suggest the large PBA voids may not match the smaller size of Li-ions.

The PBA framework as the cathode materials for lithium-ion storage was early reported in 1999^25^, the Fe_4_[Fe(CN)_6_]_3_·5.89H_2_O delivered a discharge capacity of 90 mAh g^−1^ in the first ten cycles at a constant current density of 0.1 mA cm^−2^ (lithium coin cells with 1 M LiClO_4_ in a propylene carbonate/1,2-dimethoxy ethane as electrolyte, 1:1 vol/vol). After that, there are only few research work using PBAs for lithium insertion such as KMnMn(CN)_6_, FeFe(CN)_6_^26–28^. The earliest results demonstrated poor cyclability and practicality of Li-PBA materials. Upon the rise of Na-PBA materials in recent years, there have been much attention on the compositional and structural optimization to attain better performance in SIBs, which is likely to be applied in Li-PBA cathodes. In particular, the two-electron reaction provides very appealing capacities once the M site is rationally designed for the Li_2_MFe(CN)_6_ cathodes. For example, the Li_2_FeFe(CN)_6_ has a theoretical capacity of 190 mAh g^−1^ (*vs*. 170 mAh g^−1^ for LiFePO_4_), even comparable to high-energy layered compounds (LiCoO_2_ and LiNi_0.6_Co_0.2_Mn_0.2_O_2_.). Also, the previous research works indicate less concerns of mass transfer for Na-PBAs, and similar results are expected for Li-PBAs. This is a difference for PBA materials from other Li polyanion cathodes that usually require a nano engineering process before use, because the nanoscale structural design can effectively improve ion or electron diffusion and mitigate the mechanical stress at the cathode side^29–31^. In terms of the comparison of Li-PBA and Na-PBA, the graphite anode is crucial to boost the energy density in cell-level in LIBs due to the different intercalation potentials between graphite and hard carbon^10,32^. Supplementary Fig. 1 summarizes the key parameters of lithium-based Prussian blue and other materials, highlighting the necessity to revisit the electrochemical behaviors of tailored PBA cathodes in LIBs, in particular for the water content.

In this work, we prepared lithium-containing Prussian blue hexacyanoferrate materials with different synthetic routes. The structures of samples were determined by powder neutron diffraction, and the dehydration and the chemical degradation process of Li_2−*x*_FeFe(CN)_6_ (denoted as LiFeHCF) samples were observed by operando variable temperature X-ray diffraction (XRD) and combined Thermogravimetry-infrared spectrometry (TGA-IR), confirming the influence of zeolitic water in terms of material thermal stability. Compared to other sizes of LiFeHCF samples, the LiFeHCF-1 sample with micron size and single crystal morphology exhibits a high reversible capacity of 142 mAh g^−1^ at 19 mA g^−1^. Further electrochemical evaluation shows appealing electrochemical energy storage performance of the LiFeHCF-1 sample under different tests such as high area loading (10 mg cm^−2^), high specific current (1.9 A g^−1^), long lifespan (over 1000 cycles) and wide temperature windows (−20 °C to 55 °C). The graphite||LiFeHCF-1 coin cell shows the stable cycling life with a capacity retention of 98% after 300 cycles at 25 °C, highlighting the great potentials of Prussian cathodes for practical applications in LIBs.

## Results and discussions

### Materials synthesis and characterizations

**Fig. 1:**
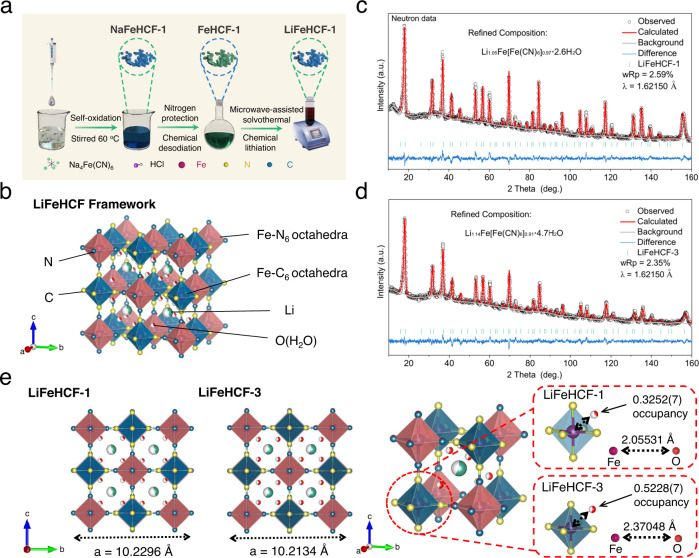
Material synthesis strategy and refinement results. **a** Schematic illustrations of the synthetic route for the LiFeHCF-1 sample. **b** Crystal structure of lithiated Prussian blue. Neutron diffraction patterns and Rietveld refinement results of the **c** LiFeHCF-1 and **d** LiFeHCF-3 samples. **e** Comparison of the unit cell parameters and coordination environment between zeolitic water and Fe(CN)_6_ octahedra for the LiFeHCF-1 and LiFeHCF-3 samples. Lithium, carbon, nitrogen, iron (coordinated with carbon), iron (coordinated with nitrogen) and oxygen atoms are shown in green, blue, yellow, purple, dark blue and red colors, respectively.

Herein, a series of LiFeHCF samples with different defects and water contents were systemically prepared by different synthetic methods. The specific methods procedures are described in the experimental section. Briefly, LiFeHCF-1, LiFeHCF-2 and LiFeHCF-3 samples were obtained through ion-exchange process, while LiFeHCF-4 and LiFeHCF-5 samples were synthesized through the self-oxidation/precipitation method. Figure 1a shows the schematic illustration of the synthetic route for LiFeHCF-1 by chemical lithiation of FeFe(CN)_6_-1 (FeHCF-1), which was first prepared via chemically extracting sodium from NaFeFe(CN)_6_-1 (NaFeHCF-1). LiFeHCF-2 and LiFeHCF-3 samples were obtained using NaFeHCF-2 and NaFeHCF-3 as the precursors that were prepared by a citrate-assisted co-precipitation method^20,22^.

To investigate the chemical desodiation/lithiation process of LiFeHCF-1, LiFeHCF-2 and LiFeHCF-3, the colors of samples at various preparation stages were recorded (Supplementary Fig. 2). Upon the chemical extraction of sodium, the blue NaFeHCF-1 and NaFeHCF-2 were turned to greenish due to the oxidation of Fe^2+^ to Fe^3+^. The blue color was recovered after microwave-assisted treatment with LiI, confirming the chemical intercalation of Li ions into the lattice. The other two LiFeHCF samples LiFeHCF-4 and LiFeHCF-5 were obtained using Li_4_Fe(CN)_6_ as both Li and Fe source (Supplementary Fig. 3). The XRD patterns confirm all LiFeHCF samples are well crystallized with the cubic structures (Supplementary Fig. 4). TGA (see Supplementary Fig. 5), element analyzer (EA) and inductively coupled plasma mass spectrometry (ICP-MS) (see Supplementary Table 1) were employed to calculate the chemical compositions of different LiFeHCF samples, the results show the LiFeHCF-1, LiFeHCF-2, LiFeHCF-3, LiFeHCF-4 and LiFeHCF-5 samples can be denoted as Li_1.05_Fe[Fe(CN)_6_]_0.97_□_0.03_·2.6 H_2_O, Li_1.36_Fe[Fe(CN)_6_]_0.96_□_0.04_·2.9 H_2_O, Li_1.14_Fe[Fe(CN)_6_]_0.91_□_0.09_·4.7 H_2_O, Li_0.63_Fe[Fe(CN)_6_]_0.96_□_0.04_·2.7 H_2_O and Li_1.0_Fe[Fe(CN)_6_]_0.90_□_0.10_·4.9 H_2_O, respectively.

To get the insights of the defect concentration and the location of water molecules, samples were further analyzed by neutron diffraction (Fig. 1b–d, Supplementary Figs. 6 and 7). The long-range ordering of Prussian Blue has been previously studied by Herren, Yusuf and others^33,34^. We built our initial model based on the Herren research work in which Fe ions are octahedrally coordinated with CN ligands in a cubic structure with a space group of *Fm-3m*. At this stage, no H_2_O molecules was put into the unit cell as their locations will be determined by the Fourier-Transform different maps after the initial refinement cycles. Constrain conditions were created such that the molar ratio of Fe and CN was consistent with the ICP result in the investigated samples. Nonetheless, the occupancy parameter was allowed to change, in addition to the parameters associated with profile, cell parameter, thermal factor, background, sample displacement, and transparency. To reduce the number of variables in the refinement, the thermal parameters of C and N were assumed to be the same. In addition, the two crystallographic distinct Fe ions were also tightened to be the same. After the above setting, all parameters were allowed to change until Rietveld analysis went to convergent. This initial step was used to determine the [Fe(CN)_6_]^3−^ vacancy concentration in the PBA structure.

Next, a Fourier-Transform neutron different map was conducted to find out the locations of coordinated and uncoordinated water molecules. It is also reported in the literature that Prussian Blue compounds comprise of various defects including [Fe(CN)_6_]^3-^ vacancies and coordinated/uncoordinated water molecules, which could affect the structures. Based on the literature^33,34^, H_2_O molecules can be placed in the CN vacancy (Wyckoff site 24e). A local maximum was also found to be located at the Wyckoff site 32f (0.366, 0.366, 0.366) which is the part of the void in the structure. O ions were then added to the N defect locations (coordinated water) and the 32 f sites (uncoordinated water) inside the unit cells. New constrains were created such that the O atoms were allowed to move between the above two sites while their total quantity was remaining constant. Once the Rietveld analysis was convergent, lithium ions position was determined by the Fourier-Transform neutron different map. Another local maximum was found to be located at the Wyckoff site 32f (0.25, 0.25, 0.25). Finally, lithium ions were added at the 32 sites in the unit cell. All of the Rietveld analysis can be convergent, and the agreement factor wRp (weighted R-factor profile) values were reasonably low (2.62%). All refinement results were summarized into Supplementary Tables 2–5. As shown in Fig. 1e, the LiFeHCF-1 sample demonstrates the lattice parameter increases from 10.2134 to 10.2296 Å as compared to LiFeHCF-3, which means higher diffusion coefficient for lithium ions^35^. In addition, the higher occupancy of water and the longer Fe–O bond distance were observed in LiFeHCF-3 samples. The water molecules located in vacancy can impede Li-ion migration, but suitable water closed to Fe(CN)_6_ octahedra may help maintain structure and avert the severe Li displacement in the <111> direction.

**Fig. 2:**
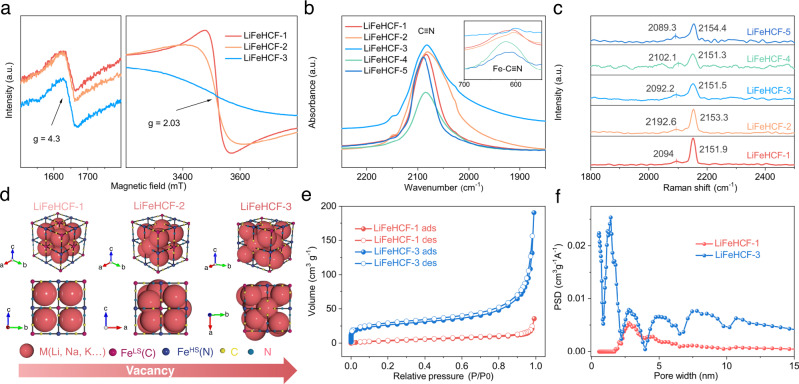
Structural characterization of different LiFeHCF samples. **a** Electron paramagnetic resonance (EPR) spectra of the LiFeHCF-1, 2, and 3 samples. **b** FTIR spectra of various LiFeHCF materials. The inset shows the enlarged plots of the spectra between 550 and 700 cm^−1^. **c** Raman spectra (1800–2550 cm^−1^) of LiFeHCF showing the evolutions of the (C≡N)^−^ group. **d** Schematic illustrations of the characteristics of LiFeHCF samples and vacancy correlations. N_2_ adsorption–desorption isotherms (**e**) and Pore-size distribution (**f**) of the LiFeHCF-1, LiFeHCF-3 samples.

Electron paramagnetic resonance (EPR) is an effective characterization technique that can be used to verify the iron species in substances, especially when studying the coordination environment and oxidation state of transition metals^36^. Figure 2a and Supplementary Fig. 8a illustrate that the EPR spectra of LiFeHCF-1, LiFeHCF-2 and LiFeHCF-3 samples have characteristic peaks at *g*-factor = 4.3 and *g*-factor = 2.03, respectively. The peak at *g*-factor = 4.3 is assigned to the four-coordinated or distorted four-coordinated isolated Fe^3+^, while the peak with *g*-factor = 2.03 was assigned to the highly symmetrical six-coordinated isolated Fe^3+^ or the characteristic peak of polymeric iron ions^37,38^. Different LiFeHCF samples show distinct coordination environment of iron bound to Fe–C_6_ octahedra or FeN_6_ octahedra. LiFeHCF-1 has a narrow peak at *g*-factor = 4.3, which is indicative of that highly symmetrical six-coordinated ferric iron ions and hence the high crystallinity and low defects. In contrast, LiFeHCF-2 and LiFeHCF-3 have relatively broad peaks at *g*-factor = 4.3, which suggests presence of the polymerized Fe ion with increased defects in lattice. The fragmented Fe(CN)_6_ group hinders Li-ion migration in the crystal structure.

The Fourier transform infrared spectroscopy (FTIR) and Raman spectroscopy (Raman) were also utilized to study the structural variation of LiFeHCF samples. Supplementary Fig. 8b shows the FTIR spectra of different LiFeHCF materials in the range of 500–4000 cm^−1^. The characteristic peaks at 494 cm^−1^ and 608 cm^−1^ are assigned to the out-of-plane bending vibration peak and the in-plane bending vibration peak of the Fe–C chemical bonds in the Fe–C≡N group, respectively^39–41^. In addition, the peaks at 1638 cm^−1^ and 3462 cm^−1^ are O–H bond stretching vibration peaks and in-plane bending vibration peaks, and the peaks at 2082 cm^−1^ represents the stretching vibration peak of the C≡N bond that is related to the transition metal ions bonded to the CN^−^ groups. Notably, the stretching vibration peak of the C≡N bond, as shown in Fig. 2b, reveals the vibrations of Fe–C≡N group of LiFeHCF samples. Compared to LiFeHCF-3, LiFeHCF-1 has a blue shift and higher peak of Fe–C≡N at 608 cm^−1^, which is attributed to the stronger C≡N chemical bond and more stable Fe–C≡N group. In Fig. 2c, Raman spectra (1800–2550 cm^−1^) show the characteristic diffraction peak attributed to the C≡N groups bonded to iron ions of different valences in the lattice^21,42^. The gradual increase of peaks at 2151 cm^−1^ and 2094 cm^−1^ indicates that the increased symmetry of the FeC_6_ and FeN_6_ octahedra, which is consistent with the EPR result. Based on the above analyses, Fig. 2d schematically illustrates the distribution of the Fe–C≡N group in LiFeHCF-1, LiFeHCF-2, and LiFeHCF-3. LiFeHCF-1 has more symmetric FeC_6_ or FeN_6_ octahedra, good crystallinity and lower defects among all samples, whereas the massively incomplete Fe–C≡N group in LiFeHCF-2 and LiFeHCF-3 possibly cause irreversible insertion/extraction of lithium ions and the increased space for zeolitic water. The different structures of LiFeHCF samples were further verified with the Nitrogen adsorption/desorption measurements and analyses. As shown in Fig. 2e, f, LiFeHCF-3 shows higher specific surface area (55 m^2^ g^−1^ vs. 15 m^2^ g^−1^) as well as more micropores (<2 nm) than for LiFeHCF-1. The high surface area is partially due to the reduced particle size of LiFeHCF-3 (see Supplementary Fig. 9), but also an indication of abundant defects resulted from the synthetic process. The Nitrogen adsorption/desorption measurements and analyses of LiFeHCF-2, LiFeHCF-4, and LiFeHCF-5 samples were also presented for comparison (Supplementary Fig. 10).

To further probe the local environment, X-ray photoelectron spectroscopy (XPS) measurements was performed with LiFeHCF samples (Supplementary Fig. 11). For high-resolution XPS spectra, the Fe *2p* peaks are located at the binding energy of 724.7 eV and 709.8 eV, corresponding to Fe^3+^ *2p*_3/2_ and Fe^3+^ *2p*_1/2_, respectively^43,44^. Within the binding energy regions of 721.2 eV and 708.4 eV, all LiFeHCF spectra show the characteristic peaks of Fe^2+^, but the signals of Fe^3+^ ions are not different from each other. The LiFeHCF-3 and LiFeHCF-5 samples have a bulging at ~712 eV caused by polymeric iron ions, which is consistent with the ERP results.

**Fig. 3:**
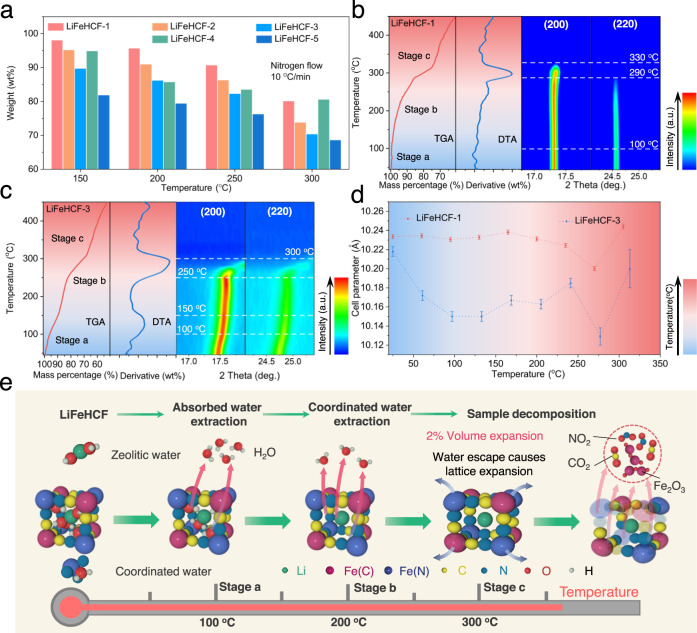
Characterizations of different LiFeHCF samples during the heating process. **a** The variation of weight in different LiFeHCF samples. Structural evolution of **b** LiFeHCF-1 and **c** LiFeHCF-3 powder samples during heating from 25 °C to 450 °C. **d** Comparison of the cell parameters changes of distorted framework of LiFeHCF samples during heating process. The error bars represent the range of cell parameters for the Prussian blue samples upon heat treatment. **e** Schematic illustration of the dehydration behavior and the chemical degradation mechanism of LiFeHCF samples.

It’s generally recognized that water content has significant influence on electrochemical performance for Prussian cathodes^10,32^. For this reason, thermogravimetric analysis (TGA) was performed to determine the presence and content of water in the lattice of LiFeHCF samples. In Supplementary Fig. 5, distinct weight loss between below 300 °C is attributed to the release of the adsorbed water and the coordinated water. The results were summarized in Fig. 3a revealing the mass loss of each LiFeHCF sample as 98.1%, 95.2%, 89.7%, 94.9%, and 81.8% at 150 °C, respectively. When the temperature was increased to 300 °C, the values were turned to 80.1%, 73.8%, 70.3%, 80.5%, and 68.6%, respectively. These results indicate less coordinated water in LiFeHCF-1 and LiFeHCF-4 samples. The more symmetric FeC_6_ or FeN_6_ octahedra in these samples lead to a decrease of water in lattice and the more stable structure; In contrast, the gradual increased defects in LiFeHCF-2, LiFeHCF-3, and LiFeHCF-5 samples lead to thermal instability, which is consistent with the previous conclusions.

The dehydration process and chemical degradation process of LiFeHCF samples were further studied with operando variable temperature XRD performed from 25 to 450 °C (Supplementary Fig. 12). Supplementary Fig. 13 is the schematic illustration of the stage with LiFeHCF powder in the heating module. Figure 3b, c (Supplementary Figs. 14 and 15) displays the TGA (DTA, differential thermal analysis) curves and the contour plots of the (200) and (220) diffraction peak of LiFeHCF-1 and LiFeHCF-3. The heating and the decomposition process can be sketched into three stages. In the first stage (stage a, from 25 °C to 100 °C), the (200) peak gradually shifts to higher angles and the (220) peak becomes weak, corresponding to the extraction of zeolitic water (absorbed water) from lattice. Then the remaining zeolitic water (absorbed water) continuously evaporate from defect sites, and the (200) and (220) peaks remain shifting and weakening from 100 °C to 250 °C/290 °C. When the temperature is increased to 300 °C/330 °C (stage b), the coordination water is deeply extracted from lattice and the samples evolve into an anhydrous phase. The disappearance of (200) and (220) peaks occurred at the 300 °C/330 °C due to the structural decomposition. We further applied the combined Thermogravimetry-infrared spectrometry (TGA-IR) technology for the LiFeHCF sample under flowing Ar protection (Supplementary Fig. 16), confirming the decomposition products include CO_2_, NO_2_ and HCN^45,46^. Significantly, the LiFeHCF-3 sample exhibits relatively broadened and weakened diffraction peaks at stage a, indicating more zeolitic water was removed from the crystal lattice, resulting in severe lattice distortion and volume reduction. Figure 3d shows more detailed comparison of the cell parameter variation during heating. Due to the release of zeolitic water and coordination water, the cell parameter is decreased from 10.218 Å to 10.129 Å for LiFeHCF-3, and the unit cell volume is contracted by 2.7%. In comparison, LiFeHCF-1 has a little shrinking of 0.9% as the cell parameters is stable from 25 °C to 290 °C (10.201 Å vs. 10.23368 Å). Afterwards, the two LiFeHCF samples exhibit the lattice expansion with volume change of ~1.2% and ~2.1% until the samples start to decompose.

Collectively, the dehydration behavior and the chemical degradation process of LiFeHCF samples is summarized in the schematic illustration of Fig. 3e. Different types of water correspond to various extraction temperatures and lattice changes. The results also suggest that the presence of zeolitic water in the crystal lattice has negative effects on thermal stability, which was in fact dominated by the increase of asymmetric Fe–C_6_/Fe–N_6_ octahedra. The correlations between thermal stability and zeolitic water content and the integrity of structural framework of LiFeHCF materials are not usually reported and discussed in the literature. The operando variable temperature XRD patterns of LiFeHCF-2 and LiFeHCF-4 show similar results as the LiFeHCF-1 and LiFeHCF-3 samples in Supplementary Figs. 17–20.

**Fig. 4:**
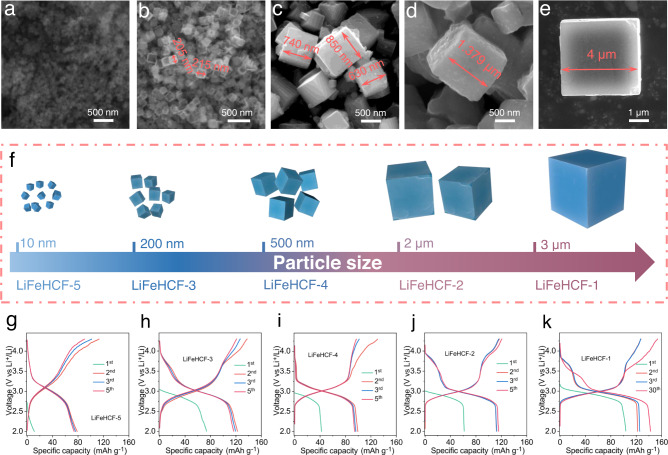
Influence of particle size on electrochemical performance of LiFeHCF samples. Particle size of **a** LiFeHCF-5, **b** LiFeHCF-3, **c** LiFeHCF-4, **d** LiFeHCF-2, and **e** LiFeHCF-1 powder samples. All images are taken from ex situ SEM measurements. **f** Schematic illustration of a microcrystalline crystallite size of LiFeHCF sample. **g**–**k** The corresponding capacity−voltage profiles of the LiFeHCF samples at specific current of 19 mA g^−1^. All LiFeHCF electrodes were evaluated in coin cell configuration using Li metal as counter electrode and tested at 25 °C.

The scanning electron microscope (SEM) images for LiFeHCF-1, LiFeHCF-2, LiFeHCF-3, LiFeHCF-4 and LiFeHCF-5 powder samples are shown in Supplementary Fig. 9. LiFeHCF samples prepared by different methods possesses similar cubic morphologies but different particles sizes (from nanometer to micrometer). In Supplementary Fig. 9a, d, the cubic features with smooth interface of LiFeHCF-1 and LiFeHCF-4 samples can be attributed to the self-decomposition synthesis methods with intrinsic slow nucleation rate (using Na_4_Fe(CN)_6_, Li_4_Fe(CN)_6_ as sodium source and lithium source, respectively). In contrast, LiFeHCF-2 and LiFeHCF-3 exhibit different morphologies with agglomerations from the other two samples, relating to the randomly aggregated process by the modified citrate-assisted co-precipitation method. Figure 4a–f shows the typical crystallite sizes of LiFeHCF samples. LiFeHCF-1 has largest size of ~ 4 μm and LiFeHCF-4 cube are only ~650–850 nm. LiFeHCF-1 sample possess a single-crystal morphology with high crystallinity, while LiFeHCF-2 and LiFeHCF-3 samples has the average crystallite sizes of ~2 μm and ~200 nm. Their precursors were synthesized by same coprecipitation method expect for using different content of sodium citrate. LiFeHCF-5 sample prepared by simple coprecipitation method (using Li_4_Fe(CN)_6_) has the smallest size of ~10 nm. All samples have different nucleation rates, and the fast crystallization may result in the presence of vacancies and the large content of zeolitic water, hindering the growth of crystallite sizes. We also attempted to directly observe the location of defects and water in the lithium-based Prussian blue lattice, but the samples too sensitive for high-resolution electron microscopy and spectroscopy, for this reason, PBA high-resolution images have rarely been reported^47^.

### Electrochemical performances of LiFeHCF samples

LiFeHCF electrodes were evaluated in coin cell configuration using Li metal as counter electrode with 1 M LiPF_6_ in ethylene carbonate (EC) and diethyl carbonate (DEC) electrolyte. Figure 4g–k shows the Galvanostatic cycling curves of LiFeHCF-1, LiFeHCF-2, LiFeHCF-3, LiFeHCF-4 and LiFeHCF-5 samples at a specific current of 19 mA g^−1^. The cells deliver discharge capacity of 126 mAh g^−1^, 117 mAh g^−1^, 122 mAh g^−1^, 103 mAh g^−1^ and 80 mAh g^−1^ in the second cycle, respectively. It is worth noting that the LiFeHCF-1 sample with the largest particle size has an activation process and the highest discharge capacity of 143 mAh g^−1^ appears after 30 cycles (Fig. 4k). Supplementary Fig. 21a give a comparison between crystallite size and electrochemical energy storage performance of LiFeHCF samples. It has been demonstrated that the crystallite size is closely related to the electrochemical behaviors of potassium Prussian white materials (K_1.7_Fe[Fe(CN)_6_]_0.9_)^48^. The K-based Prussian blue sample with a small size (~20 nm) shows the best electrochemical performance; however, the micron-sized Prussian blue samples seem more suitable for lithium-ion batteries.

**Fig. 5:**
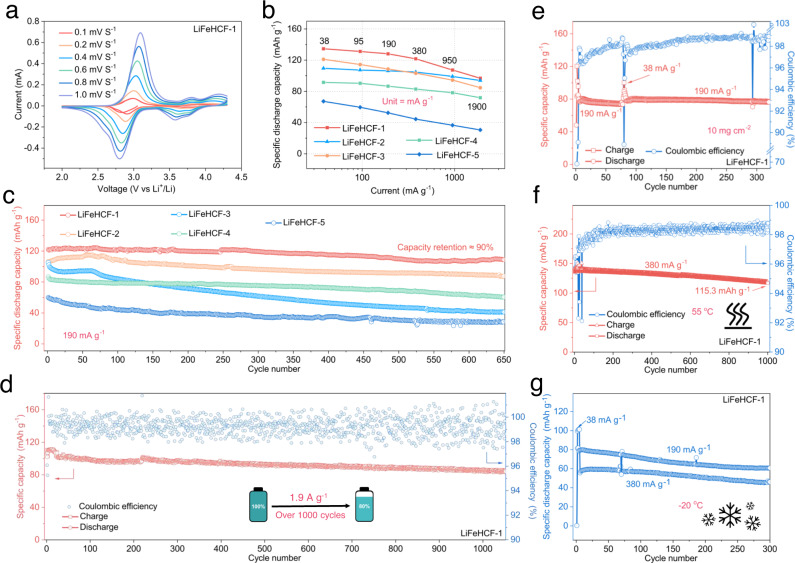
Electrochemical evaluation of LiFeHCF-1 samples. **a** Cyclic voltammetry curves of LiFeHCF-1 sample at different scanning rates. **b** Rate performance of LiFeHCF samples at various specific currents. **c** Cycling performance of LiFeHCF samples at 190 mA g^−1^. **d** Long-term cycling of LiFeHCF-1 material at 1.9 A g^−1^. **e** High-loading performance (10 mg cm^−2^). **f**, **g** High and low temperature tests at 55 °C and −20 °C. All LiFeHCF electrodes were evaluated in coin cell configuration using Li metal as counter electrode and tested at 25 °C (except where differently indicated). The specific capacity values were calculated base on the mass of positive electrode active material.

The typical discharge voltage plateau at 3.1 V can be observed for all samples, but the LiFeHCF-1, LiFeHCF-2 also have a second plateau at 3.8 V, which correlate with the reduction reactions Fe^3+^/Fe^2+^ couples during the lithiation process. Cyclic voltammetry (CV) test was performed to understand the reasons for various voltage plateau of LiFeHCF samples. As shown in Fig. 5a, the cathodic peaks at 3.1 V belongs to the redox reactions of high-spin Fe^3+^/Fe^2+^ couples coordinated with nitrogen, and the high-potential peaks at 3.8 V are related to the low-spin Fe^3+^/Fe^2+^ couples coordinated with carbon^49^. The CV curves of other LiFeHCF samples under the same test conditions are shown in Supplementary Fig. 21b–d. The peaks do not appear in high voltage range for LiFeHCF-3, indicating there is correlation between Fe–C_6_ octahedra and redox potentials upon lithium ions insertion/extraction.

Rate capability of LiFeHCF electrodes were furthered investigated in non-aqueous Li metal coin cell configuration as shown in Fig. 5b and Supplementary Fig. 22. Li metal cells with LiFeHCF-1-based positive electrodes show the best performance among all samples. The capacities are 135, 131, 128, 122, 107, and 97 mAh g^−1^ at 38, 95, 190, 380, 950, and 1900 mA g^−1^, respectively. Also, the LiFeHCF-1 electrode demonstrates superior cycling performance at 190 mA g^−1^, delivering a reversible capacity of 109 mAh g^−1^ and good retention of ~90% after 650 cycles. In comparison, the capacities of LiFeHCF-2, LiFeHCF-3, LiFeHCF-4, and LiFeHCF-5 electrodes at 190 mA g^−1^ are 87, 41, 61, 29 mAh g^−1^ and capacity retention of 82.3%, 40%, 70%, and 47.4%, respectively (Fig. 5c). Significant capacity fading occurs after 100 cycles for LiFeHCF-3 and LiFeHCF-5. The poor stability is possibly related to the large number of vacancies that hinder Li-ion migration through the <100> direction. Besides, the zeolitic water in the structures is gradually extracted during the charge/discharge process, further accelerating electrolyte decomposition and reducing coulombic efficiency. The LiFeHCF-1 electrode exhibits noticeable long-term cycling performance at a high specific current of ~ 1.9 A g^−1^. As shown in Fig. 5d, the cell deliveres a discharge capacity of 118.9 mAh g^−1^ at the initial cycle and maintains 80.7% capacity over 1000 cycles (0.019% capacity fading per cycle) with a high coulombic efficiency of 99.3%. Even positive electrodes with a mass loading of 10 mg cm^−2^, the Li metal coin cell has a capacity retention of 98% after 300 cycles at 190 mA g^−1^ (Fig. 5e); it also shows good performance at 55 °C and −20 °C (Fig. 5f and g). Supplementary Table 6 summarizes the electrochemical performances of representative Na-PBA cathodes and our LiFeHCF, highlighting its potentials for the application in LIBs.

To better evaluate the relation between discharge and lithium-ion diffusion properties, galvanostatic intermittent titration technique (GITT) measurement was performed by inserting Li^+^ into LiFeHCF samples at a specific current of 190 mA g^−1^ between 2.0 and 4.3 V, and the cell was discharged from 4.3 V for 10 min followed by a rest of 120 min. As shown in Supplementary Fig. 23, the sloping regions at 3.8–3.5 V and 2.7–2.0 V are indicative of solid solution process, while the overpotential between 3.2–2.7 V (corresponding to lithium insertion from Li_0.5_FeFe(CN)_6_ to Li_1.8_FeFe(CN)_6_) and the flat sloping region indicates two-phase mechanism of the insertion of more lithium^49,50^.

### Investigation of phase transitions of LiFeHCF-1 sample during cycling

**Fig. 6:**
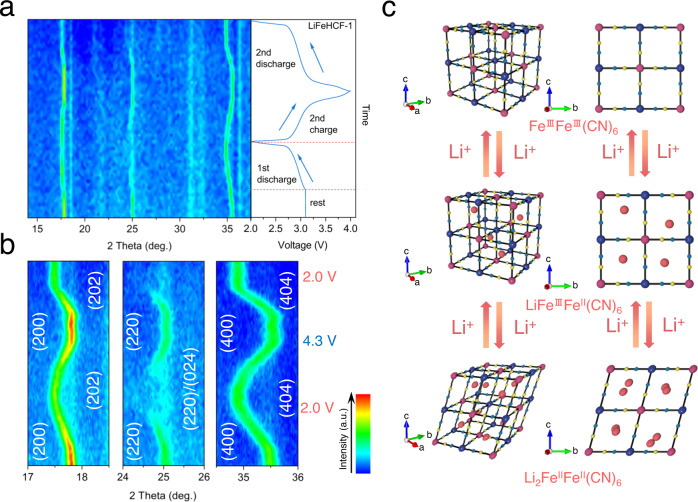
Structural evolution of the LiFeHCF-1 sample during charge and discharge. **a** Operando XRD patterns. **b** Detailed information of the (200), (220)/(024), and (400) diffractions. **c** Schematic of the phase evolutions upon Li insertion/extraction. The Operando battery for XRD testing was carried out by stainless steel cell with beryllium window at specific current of 9.5 mA g^−1^ at 25 °C.

Operando XRD was conducted to study the phase transitions during Li^+^ insertion and extraction in the LiFeHCF-1 sample. The digital image and schematic illustrations of the operando XRD cell and instrument are shown in Supplementary Fig. 24a, b. In Fig. 6a, the peaks at 17.8° and 35.5° are gradually shifted to lower angles and the peak at 25° is disappeared (Supplementary Fig. 24c), indicating the increases of the volume of the unit cell on lithiation. The peaks are recovered at the initial position after 2nd discharge, suggesting the formation of Li-rich Li_1+x_FeFe(CN)_6_. It is worth mentioning that this phenomenon is different from the observation of irreversible lattice shrinking with Na Prussian blue materials^50^. The cubic unit cell and the ease of movement of lithium ions for LiFeHCF-1 material have a highly reversible phase transition that helps long cycle life (over 1000 cycles at 1.9 A g^-1^).

The expanded views of the (200), (220), and (400) peak regions are shown in Fig. 6b. The (200) peak gradually shifts from 17.8° to 17.52° upon the first discharge process, which indicates the evolution of the cell parameters during the second lithium intercalation process. The full insertion of Li^+^ to Li_1+*x*_ FeFe(CN)_6_ lattice evolved a new phase when the voltage reached 2.0 V. The subsequent charge shows the recovery of peak, corresponding to the deintercalation of Li^+^ vs. Li_2−*x*_FeFe(CN)_6_ and FeFe(CN)_6_. Notably, the (200) and (400) diffraction peaks are recovered after re-discharged to 2.0 V, confirming the high reversibility of the Li_2−*x*_FeFe(CN)_6_ phase during Li intercalation/dentercalation. This highly reversible Li migration process is likely to be attributed to the more symmetric Fe–C_6_/Fe–N_6_ octahedra of the LiFeHCF-1 sample. Also, the (220) diffraction at 25° splits into two peaks after discharged to 2.0 V, which is another evidence of high lithiation degree of the LiFeHCF-1-containing positive electrode. As schematically illustrated in Fig. 6c, it is assumed that the onset of Li diffusion is through the <100> direction for the cubic Fe^III^Fe^III^(CN)_6_. Upon the subcube sites are half-occupied, the diffusion path is changed to the <111> direction due to the volume expansion accompanied by the lattice transformation from cubic LiFe^III^Fe^II^(CN)_6_ to rhombohedral structure Li_2_Fe^II^Fe^II^(CN)_6_, which is in agreement with the GITT results.

### Lithium-ion and water molecules diffusion behavior

**Fig. 7:**
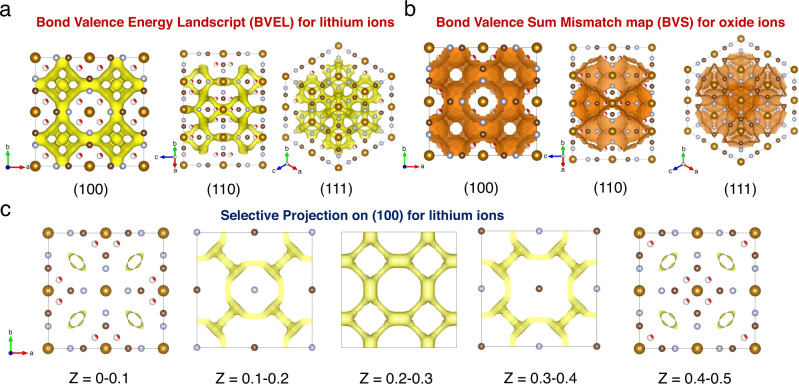
Theoretical prediction of the pathways of Li-ions for PBAs. **a** Bond Valence Energy Landscape (BVEL) calculation for Li-ions in LiFeHCF samples. **b** Bond Valence Sum Mismatch map (BVS) calculation for oxide ions in LiFeHCF samples. The selected projection on (100), (110) and (111) planes were presented, indicating that Li-ions can be transported 3-dimensionally through the void of PBA compounds. **c** BVEL with different *Z* values on the (100) planes.

To understand the Li-ion diffusion behaviors, bond valence energy landscape (BVEL) calculations was performed with LiFeHCFs. Since the C and N atoms are covalently bonded together in the CN ligands, the correct charge on C and N could not be easily determined. Nonetheless, we arbitrarily assigned C and N with three different scenarios: 1. C4+ and N3−, and 2. C3+ and N3−, and 3. C4+ and N3−. We expect that the exact energy barrier would be estimated with uncertainty. However, such a method could still be useful to illustrate the topology of the pathways of lithium ions. As shown in Fig. 7a, we reveal that Li-ion diffusion can proceed through the a 3.2 × 2.0 Å^2^ ellipsoid pore window centered at (0.25, 0.25, 0) into the 3D-interconnected cubic 3 × 3 × 3 Å^3^ nano-cage centered at (0.25, 0.25, 0.25). The diffusion of lithium ions was mainly driven by two factors: 1. the columbic interaction between the negative charged framework and the position of the charge on the lithium ions when the Fe ions were undergone reduction reaction to change their valence states from 3+ into 2+, upon the discharging process; 2. the chemical potential different between the PBA compounds and its surrounding environment. In Supplementary Fig. 25, since the occupancy of H_2_O molecules is 0.32 at the 32f site (0.25, 0.25, 0.25) in the unit cell, the probability to H_2_O molecule at this site was 32%. The topological pathway was very similar to the simulation without putting an addition water molecule in the unit cell (Supplementary Figs. 26, 27 and Table 7). However, the density of the percolation network was found to shrink significantly, as we compared the same bond valence mismatch value of 1. This indicated that the water molecule can reduce very significantly on the conductivity of the lithium ions in the unit cell. If the water content is increased in the framework, the apparent percolation network will shrink significantly. Therefore, the lithium-ion conductivity will be dropped dramatically.

Recently, a few computational studies have tried to explain how water molecules can stabilized the structure of PBAs upon electrochemical cycling^51,52^. This enlighten us to further investigate diffusion behaviors of water molecules in PBAs as water molecules could be exchanged between the non-aqueous electrolyte solution and the PBA structure during the charge/discharge processes. As shown in Fig. 7b, c, the BVS mismatch map on PBA indicates the diffusion pathway of water molecules are resemble but wider spread behavior as the Li-ion percolation network. This suggests that water molecules could easily block the diffusion pathway of Li-ions if their concentration is high (>1.8 mol% or 9.8 wt.%). Therefore, minimizing the water content and the defects in PBAs could help to optimize the electrochemical energy storage performances of Li-based cells with PBAs-containing positive electrodes.

As shown in Supplementary Fig. 28, additional supercell analysis was performed to enlarge the unit cells along x by 2 and along y by 2 to provide better understanding on the effect of H_2_O molecules in space as they can either occupy or un-occupy into the 32f sites. Of 32 sites in the supercells, 10 of them will be filled with H_2_O molecules. This will come with a total combination of 64512240. Thanks to the supercell program, the columbic energies of all supercells can be calculated and ranked based on their energies for outputting. Below is the additional BVEL analysis on the lowest energy supercell to show the effect of H_2_O on the lithium-ion diffusion property. First, it is interesting that water molecules are not evenly distributed inside the supercell, while some of them like to aggregate to form water clusters. With a small value of BVEL, 3D percolation network can be observed but they are formed around the water clusters. As we progressively increase the BVEL values, lithium ion diffusion network can also form as they pass through the void where water molecules are absent. Interestingly, the increase in the density of 3D percolation network is mainly due to the new pathways with void, while the original pathways, with water clusters nearby, is hardly changed. Therefore, we can conclude that an excess of water molecules will decrease the over density of lithium-ion percolation network in the structure.

### Electrochemical performance of LiFeHCF-1-based positive electrodes in Li-ion coin cell configuration

**Fig. 8:**
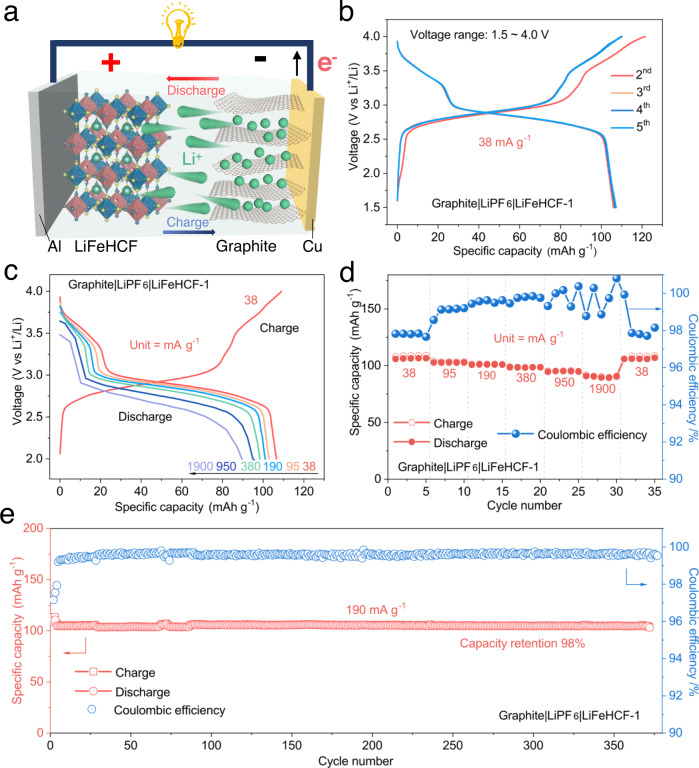
Electrochemical performance of LiFeHCF-1-based positive electrodes coupled with graphite-based negative electrodes. **a** The schematic illustration of the graphite | |LiFeHCF full cell. **b** Galvanostatic charge–discharge voltage profiles of the full-cell at 38 mA g^−1^. Voltage profiles (**c**) and rate capability (**d**) at different current densities from 38 mA g^−1^ to 1900 mA g^−1^. **e** The demonstration of long-term cycling of the cell at 190 mA g^−1^. All LiFeHCF graphite||LiFeHCF full cell were tested at 25 °C and the specific capacity values were calculated based on the mass of positive electrode active material.

To further validate the practical application of the LiFeHCF-1 cathode, Li-ion cells were assembled and tested with commercial graphite as the negative electrode active material (Fig. 8a and Supplementary Fig. 29). At a specific current of 38 mA g^−1^ (considered the mass of positive electrode active material), the graphite||LiFeHCF−1 coin cell gave a reversible capacity of 106 mAh g^−1^ (Fig. 8b) with an average discharge voltage around 2.9 V, no capacity attenuation was appeared after 5 cycles over the voltage range of 1.5 ~ 4.0 V. In Fig. 8c, d, the full cell showed good reversible capacity 104, 102, 99, 95 mAh g^−1^ at different rates of 95 mA g^−1^, 190 mA g^−1^, 380 mA g^−1^ and 950 mA g^-1^, respectively, and the specific capacity could be recovered to 109 mAh g^−1^ at specific current of 38 mA g^−1^. Interestingly, the cell can be cycled up to 1.9 A g^−1^ delivering a specific discharge capacity of about 90 mAh g^−1^. Furthermore, Fig. 8e shows a high capacity of 105 mAh g^−1^ and capacity retention of 98% (average coulombic efficiency ≈ 99.6%) over 370 cycles.

In summary, we systematically studied the preparation of various LiFeHCF samples and compared their Li-ion storage performance in a non-aqueous environment. The impacts of zeolitic and crystal water in terms of thermal stability and structural framework integrity have been revealed by using advanced technologies including powder neutron diffraction, thermogravimetry-infrared spectrometry, operando variable temperature X-ray diffraction and Bond Valence Energy Landscape calculations. The tailored LiFeHCF material with micron size and single crystal morphology exhibits high capacity of >140 mAh g^−1^ (at 19 mA g^−1^) and long lifespan over 1000 cycles, as well as appealing performance under practical conditions such as high loading (10 mg cm^−2^), wide temperature (−20 to 55 °C) window and Li-ion cell configuration (graphite||LiFeHCF).

## Methods

### Materials synthesis

LiFeHCF-1, LiFeHCF-2 and LiFeHCF-3 were prepared by the ion-exchange method from NaFeHCFs^53,54^. Typically NaFeFe(CN)_6_−1 (NaFeHCF-1) was first obtained via self-oxidation/precipitation^55,56^. First, 2 mmol sodium ferrocyanide (Na_4_Fe(CN)_6_, 99.7%, Aladdin) were dissolved in 100 ml deionized water to afford clear solution under stirring (400 rpm). Next, 1 mL of hydrochloric acid (37 wt% solution in water, Acros) was added dropwise to the solution, and then the solution was heated at 60 °C under vigorous stirring (600 rpm) for 4 h. The resulting dark blue suspension was collected by centrifugation (8000 rpm, 5 min), washed with water (40 ml) and ethanol (40 ml) three times, and dried in vacuum oven at 100 °C for 24 h to obtain NaFeHCF-1. FeFe(CN)_6_-1 (FeHCF-1) was prepared by chemical desodiation using tetrafluoroborate (NO_2_BF_4_, 96%, Alfa, excess 50%) under nitrogen atmosphere with ultra-dry acetonitrile medium (99.9%, water ≤ 30 ppm, Innochem). LiFeFe(CN)_6_-1 (LiFeHCF-1) was synthesized by chemical lithiation through a microwave-assisted solvothermal (MW-ST) process (using Anton Paar Monowave 400, 600 rpm) at 80 °C. LiI (99.9% metals basis, Aladdin) was served as both lithium source and reducing agent. The obtained sample were collected by centrifugation (8000 rpm, 5 min), washed by acetonitrile anhydrous and vacuum-dried 12 h at 100 °C.

The LiFeHCF-2 and LiFeHCF-3 samples were prepared by the same route except for the different NaFeHCF-2 and NaFeHCF-3. Here both NaFeHCF samples were synthesized by a modified citrate-assisted co-precipitation method^20,57^. Briefly, 6 mmol iron sulfate heptahydrate (FeSO_4_·7H_2_O, 99.5%, Acros) and 25 g (NaFeHCF-2) or 5 g (NaFeHCF-3) of sodium citrate (C_6_H_5_Na_3_O_7_, 98%, Aladdin) were dissolved in 100 mL of deionized water, stirring (400 rpm) until a clear solution A was formed. 4 mmol Na_4_Fe(CN)_6_ was added to 100 mL deionized water, labeled as solution B. Then, solution A was slowly added to solution B under vigorous stirring (600 rpm) and a milky white precipitate formed immediately. The mixture was stirred 12 h, and the product were collected by centrifugation (8000 rpm, 5 min), washed by deionized water (40 ml) and ethanol (40 ml) and vacuum-dried 24 h at 100 °C.

LiFeFe(CN)_6_-4 and LiFeFe(CN)_6_-5 (LiFeHCF-4 and LiFeHCF-5) samples were prepared using lithium ferrocyanide (Li_4_Fe(CN)_6_). Li_4_Fe(CN)_6_ samples was synthesized following the same methodology as reported in previous literature^58,59^. Briefly, 56 mmol potassium ferrocyanide (K_4_Fe(CN)_6_, 99%, Acros) was dissolved in 25 ml deionized water, and 14 mmol lithium perchlorate (LiClO_4_, 99.99%, Aladdin) solution were mixed with continuous magnetic stirring (400 rpm). A white precipitate of potassium perchlorate was formed, which was removed by centrifugation (8000 rpm, 3 min), while the solution was heated to 70 °C to remove partial water. Next, the obtained solution was placed in a refrigerator overnight to allow potassium perchlorate was precipitated. The above steps were repeated several times until no KClO_4_ precipitation was observed. The Li_4_Fe(CN)_6_ was dried at 150 °C. LiFeHCF-4 and LiFeHCF-5 was prepared by the same method as NaFeHCF-1 and NaFeHCF-3 except used Li_4_Fe(CN)_6_ without citrate used during the synthesis.

### Materials characterizations

The obtained LiFeHCF samples were investigated by powder XRD analysis (Rigaku Miniflex 600) equipped using Cu kα radiation at 40 kV and 20 mA. The crystal structure of LiFeHCF samples was analyzed by neutron diffraction. The diffraction data (LiFeHCF-1, 3 and 4 samples) was collected at the ECHIDNA high-resolution powder diffractometer with a monochromatic wavelength of 1.6215 Å in Australian Nuclear Science and Technology Organization (ANSTO). TOF neutron diffraction measurement of LiFeHCF-2 was conducted at Multiple Physics Instrument (MPI) in China Spallation Neutron Source (CSNS). 2–3 g of powders were put into vanadium cans and the measurement time was about 3 h for each sample. The diffraction data was subsequently analyzed by GSAS EXPGUI^60^. The content of Li, Fe, C, N elements in the LiFeHCF samples were identified by ICP analysis (OPTIMA 8000DV Optical Emission Spectrometers) and Element analyzer (Vario EL Cube), the relative standard deviation of the measured samples was less than 1.5%. Scanning electron microscopy (SEM) images were performed by FEI Verios 460 L. X-ray photoelectron spectroscopy (XPS) was taken on an ESCALAB250Xi (Thermo Scientific) spectrometer equipped with X-ray source (*hv* = 1486.6 eV, monochromatic Al Ka, 150 W). Fourier transform infrared (FTIR) spectra were carried out on a Fourier transform infrared spectrometer (Perkin Elmer) with KBr disk method. Raman spectra were measured at a Raman microscope with a 325 nm excitation laser (HORIBA JOBIN YVON S.A.S.). Nitrogen adsorption/desorption measurements were performed on Autosorb-iQ-MP (Quantachrome), the micropore surface areas, pore size distribution and pore volumes were determined from nitrogen (N_2_) adsorption branch at 77 K with Brunauer–Emmett–Teller (BET) theory and the Barrett–Joyner–Halenda (BJH) model. EPR spectra were recorded from Bruker EMXplus-6/1 EPR spectrometer (9065.8 MHz, X band, 300 K). Thermogravimetry coupled with infrared spectrometry (TGA-IR) techniques (Perkin Elmer, Frontier Mid-IR FTIR/STA6000-TL9000-Clarus SQ8) was employed to confirm the decomposition products. Operando variable temperature X-ray diffraction measurement was performed on Rigaku Miniflex 600 from 25 °C to 450 °C, the powders sample was placed in heating module (Supplementary Fig. 13).

### Electrochemical measurements

The LiFeHCF electrodes slurry were coated from N-methyl-2-pyrrolidone (NMP, 99.9%, Aladdin) onto aluminum collectors using polyvinylidene fluoride (PVDF, 99.9%, Arkema) as the binder. The typical electrode was made with a formula of active material (LiFeHCF powder): Super P (99.9%, Lion corporation): PVDF = 70:20:10. Then the electrodes were vacuum dried at 100 °C for 12 h and the active mass loading was ~1.5 mg cm^−2^. CR2032 lithium coin cells were fabricated inside an Ar-filled glovebox (H_2_O < 0.1 ppm, O_2_ < 0.1 ppm) with 1 M LiPF_6_ in diethyl carbonate (DEC) and ethylene carbonate (EC) (1:1 vol/vol) electrolyte (water content <10 ppm), a metallic lithium (0.45 mm, 99.9%, Innochem) negative electrode, and celgard separator (25 μm, 55% porosity, 0.064 μm pore size). The charge-discharge test was carried out on Land CT2001A battery cycler and tested in constant temperature test room (25 ± 1 °C, except where differently indicated). The high and low-temperature tests were investigated on the high-low temperature test chamber (SHIPAC, operating temperature range: - 75 °C~150 °C ± 1 °C). The specific capacity was obtained based on the mass of positive active material and at least two parallel cells were tested for individual electrochemical experiment. Cyclic voltammetry (CV) tests were conducted on an Ivium electrochemical Workshop between 2.0–4.3 V vs. Li/Li ^+^ at various scan rates of 0.1 mV s^−1^, 0.2 mV s^−1^, 0.4 mV s^−1^, 0.6 mV s^−1^, 0.8 mV s^−1,^ and 1.0 mV s^−1^. GITT testing of coin cell at 9.5 mA g^−1^ between 2.0–4.3 V, in which the cell was discharged for 10 min followed by 120 min resting. The Operando XRD test was carried out with a stainless steel electrochemical cell with beryllium window at 9.5 mA g^−1^ from 2.0 to 4.0 V, which was assembled with metallic lithium negative electrode, Celgard separator, and positive electrode. The full cell was assembled with commercial graphite (50~90 μm, 99.7%, Shenzhen Kejing Star) as the anode, and the graphite electrodes were made by commercial graphite (90 wt%) and PVDF (10 wt%). The loading mass of LiFeHCF and graphite was ~1.5 mg cm^−2^ and the LiFeHCF|Graphite mass ratio is 1:0.8. The slurry in NMP was coated on copper foil (9 μm, 99.8%, Shenzhen Kejing Star) and dried under vacuum at 120 °C.

### Calculation method

The lithium-ion migration behavior was estimated by 3DBVSMAPPER. In particular, Bond Valence Energy Landscapist (BVEL) was calculated by lithium-ion migration pathways in two situations: 1. Valence states of C, N, O, and Fe were assigned as +2+, +3, −2 and +3; 2. Valence states of C, N, O and Fe were assigned as +4, +3, −2, and +3, respectively. The two calculated percolation networks were very similar with each other, indicating the deviation of valence state for C between 2 and 4 was not sensitive to the diffusion pathway. Similarly, the water diffusion pathway was also analyzed by the same methods. Two Valence states of C, N, O were separately considered as +4, +3, −2 and +2, +3, −2, respectively. The diffusion percolation networks were found to be non-sensitive with the valence states of C and N atoms.

## Supplementary information

Supplementary Information

## Data Availability

The data that support the findings of this study are available from the corresponding author upon reasonable request. Source data are provided with this paper.

## Source data

Source Data
