# Supplementary material for: Lithiated Prussian blue analogues as positive electrode active materials for stable non-aqueous lithium-ion batteries
Source: Nat Commun. 2022 Dec 16;13:7790. doi: 10.1038/s41467-022-35376-1 (PMC9758126; doi:10.1038/s41467-022-35376-1)
Supplement: Supplementary file 1 — Supplementary Information [file 41467_2022_35376_MOESM1_ESM.pdf]

Supplementary Information

Lithiated Prussian blue analogues as positive electrode active materials for stable non-aqueous lithium-ion batteries

Ziheng Zhang[1,2], Maxim Avdeev[3], Huaican Chen[4,5], Wen Yin[4,5], Wang Hay Kan[4,5]*, and Guang He[1,6]*

[1] *Tianjin Key Laboratory of Advanced Functional Porous Materials, Institute for New Energy Materials and Low-Carbon Technologies, School of Materials Science and Engineering, Tianjin University of Technology, Tianjin 300384, China*

[2] *Renewable Energy Conversion and Storage Center (RECAST), Haihe Laboratory of Sustainable Chemical Transformations, Key Laboratory of Advanced Energy Materials Chemistry (Ministry of Education), College of Chemistry, Nankai University, Tianjin, 300071, China*

[3] *Australian Nuclear Science and Technology Organization (ANSTO), Lucas Heights, New South Wales 2234, Australia*

[4] *Spallation Neutron Source Science Center, Dalang, Dongguan 523803, China*

[5] *Institute of High Energy Physics, Chinese Academy of Sciences, Beijing, 100049, China*

[6] *Tianneng Co. Ltd, Huzhou 313100, China*

*Corresponding authors: heguang@tjut.edu.cn (Guang He);

jianhx@ihep.ac.cn (Wang Hay Kan).

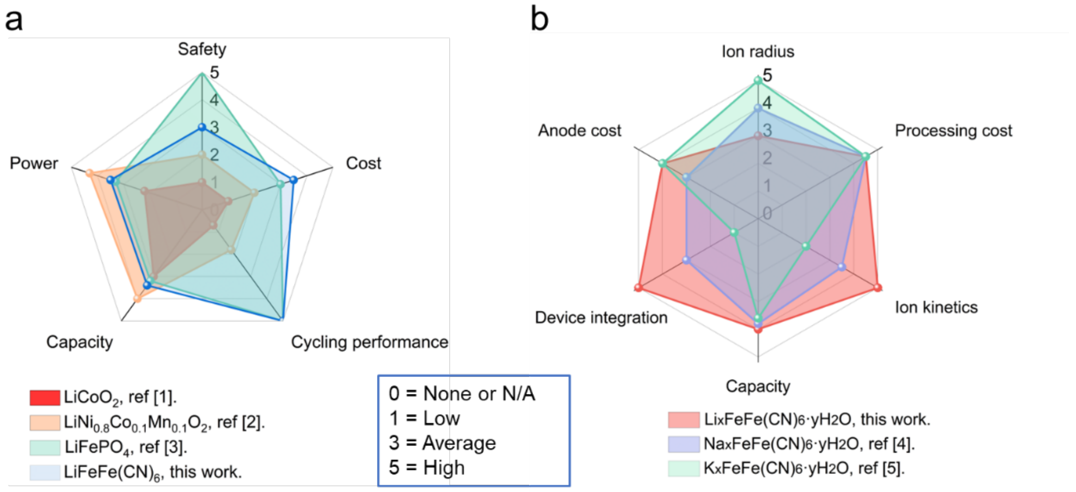

Supplementary Figure 1. (a) Multifactorial comparison of different properties of various Li-ion battery positive electrode active materials: capacity, power, safety, cost (a low value corresponds to high cost of production) and cycling performance. (b) Multifactorial comparison of different properties of various Prussian blue analogue active materials for Li-, Na- and K- ion storage.

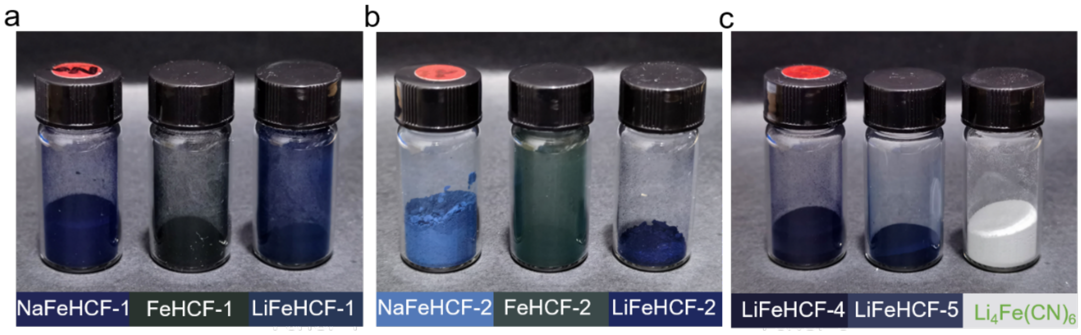

Supplementary Figure 2. Sample colors of (a) LiFeHCF-1, (b) LiFeHCF-2. (c) LiFeHCF-4 and LiFeHCF-5 powders during the synthesis process.

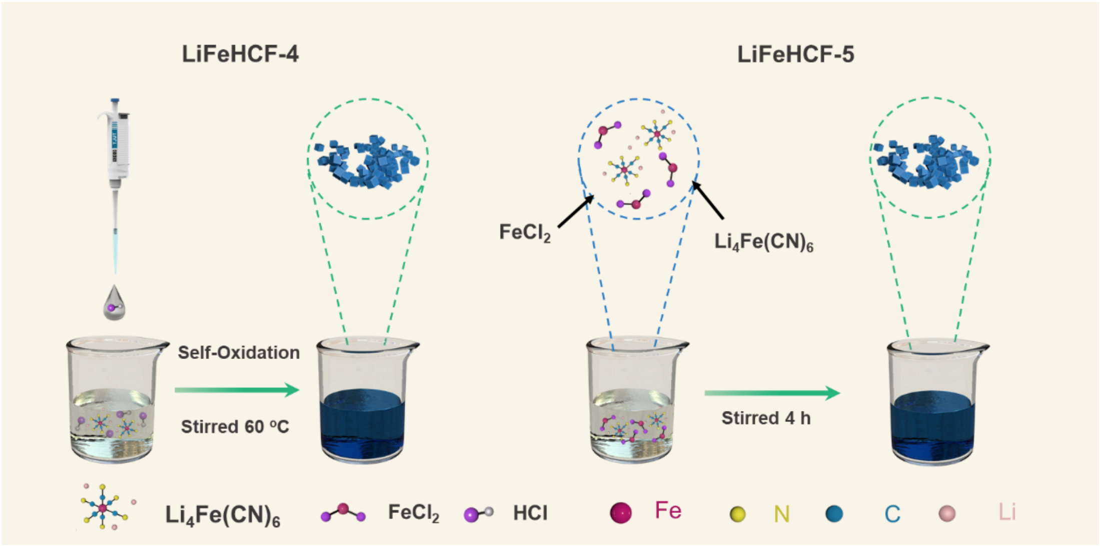

Supplementary Figure 3. Schematic illustrations of the synthetic process of LiFeHCF-4 and LiFeHCF-5 samples.

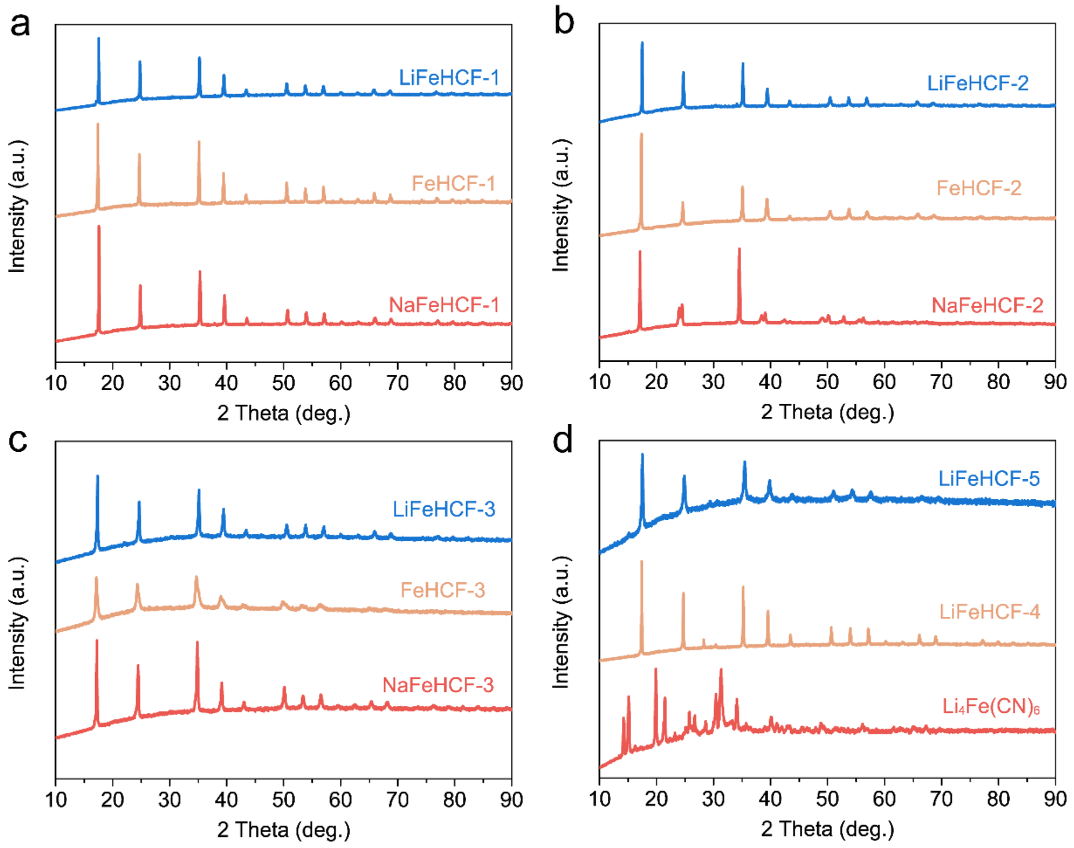

Supplementary Figure 4. Powder XRD patterns of (a) LiFeHCF-1, (b) LiFeHCF-2, (c) LiFeHCF-3, (d) LiFeHCF-4 and LiFeHCF-5 materials and precursors.

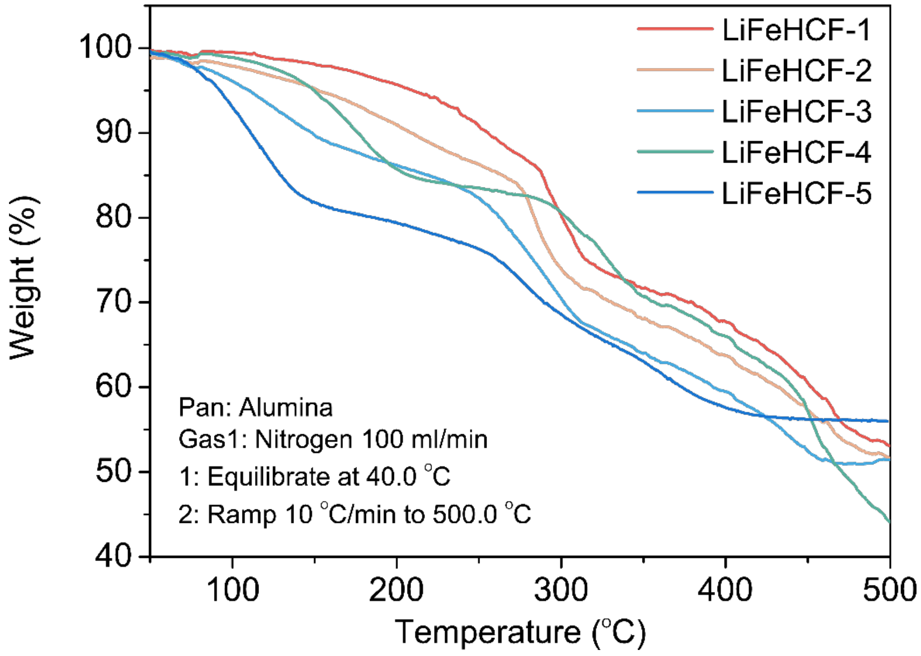

Supplementary Figure 5. TGA curves of different LiFeHCF samples.

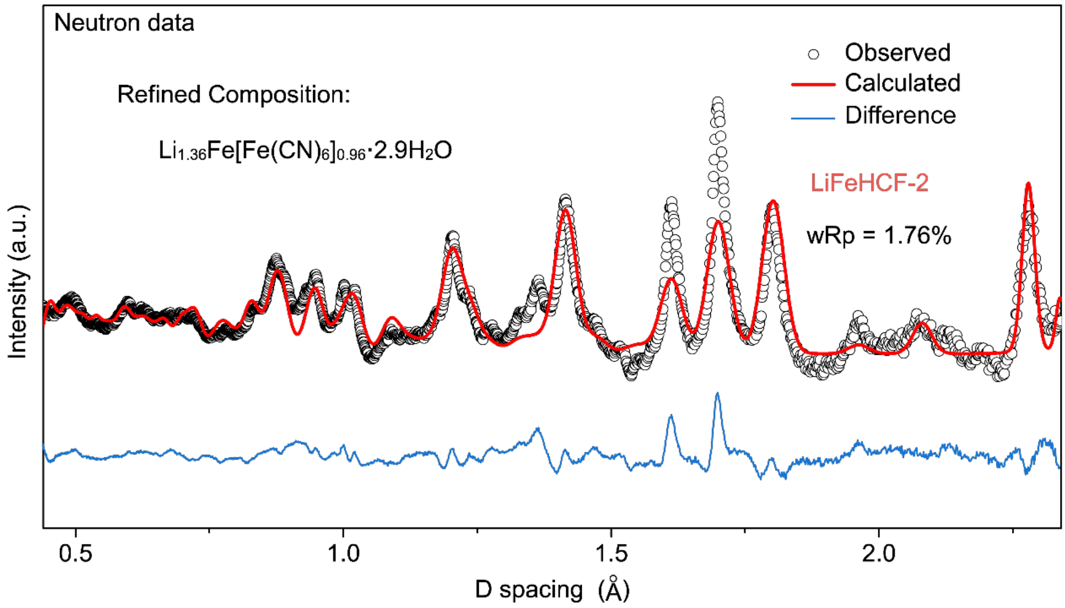

Supplementary Figure 6. Neutron diffraction pattern and Rietveld refinement results of the LiFeHCF-2 powder sample.

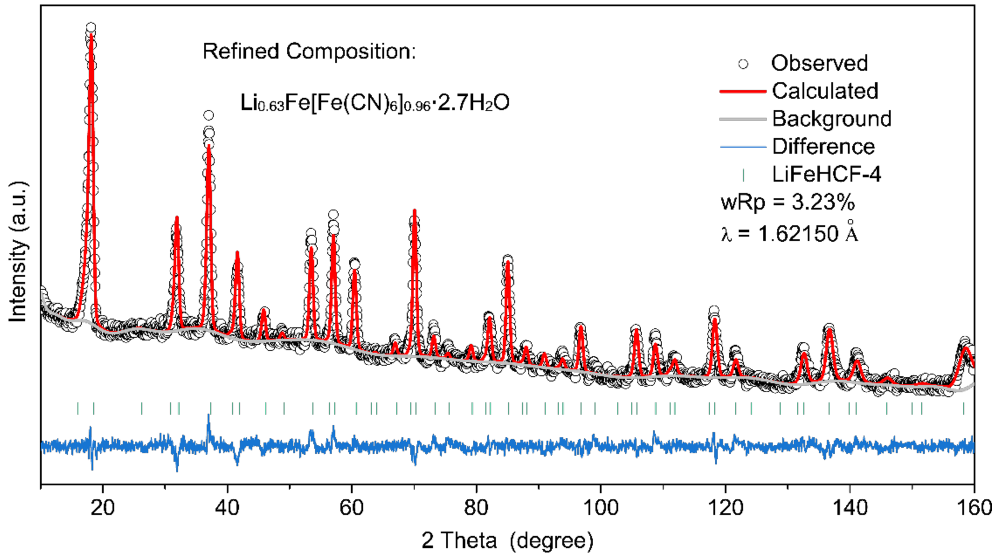

Supplementary Figure 7. Neutron diffraction pattern and Rietveld refinement results of the LiFeHCF-4 powder sample.

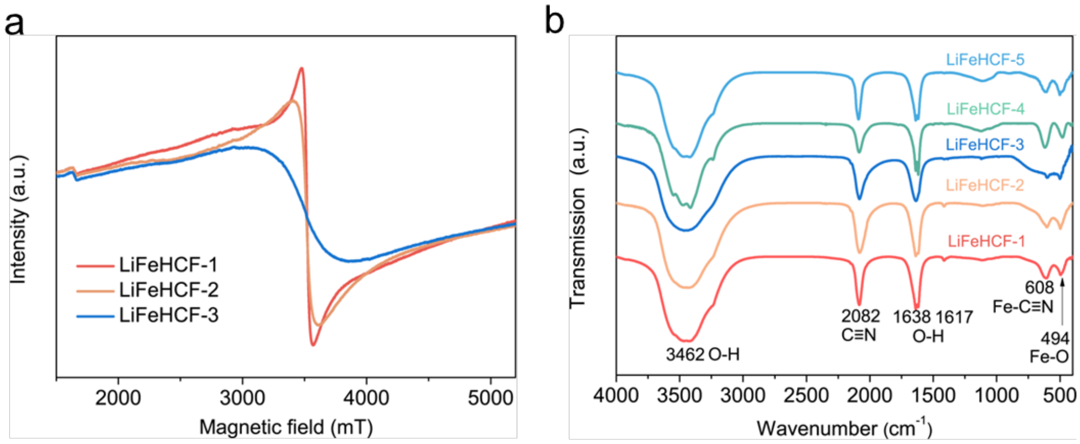

Supplementary Figure 8. Structural characterizations of different LiFeHCF powder samples. (a) EPR spectra, (b) FTIR spectra.

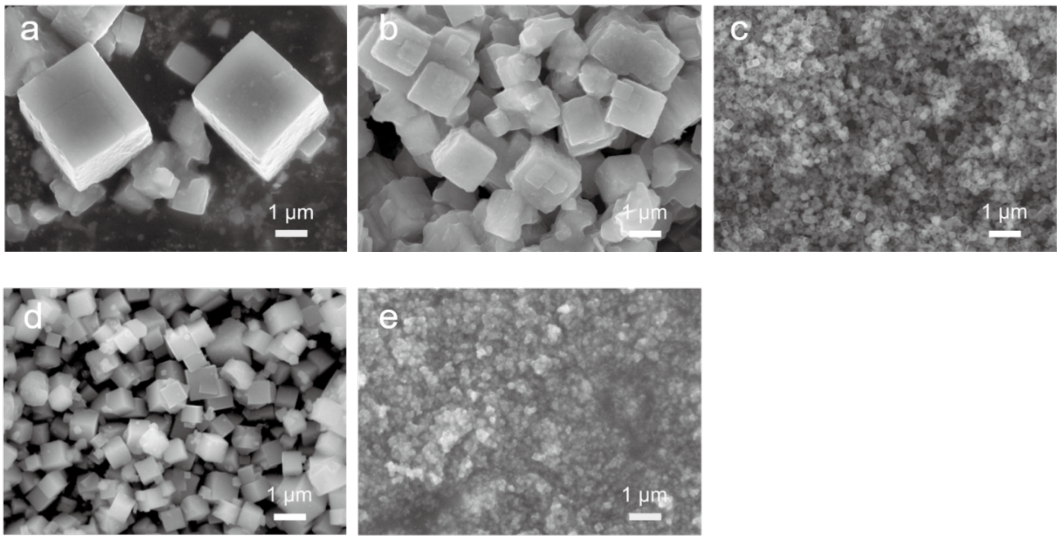

Supplementary Figure 9. SEM images of (a) LiFeHCF-1, (b) LiFeHCF-2, (c) LiFeHCF-3, (d) LiFeHCF-4 and (e) LiFeHCF-5 powder samples.

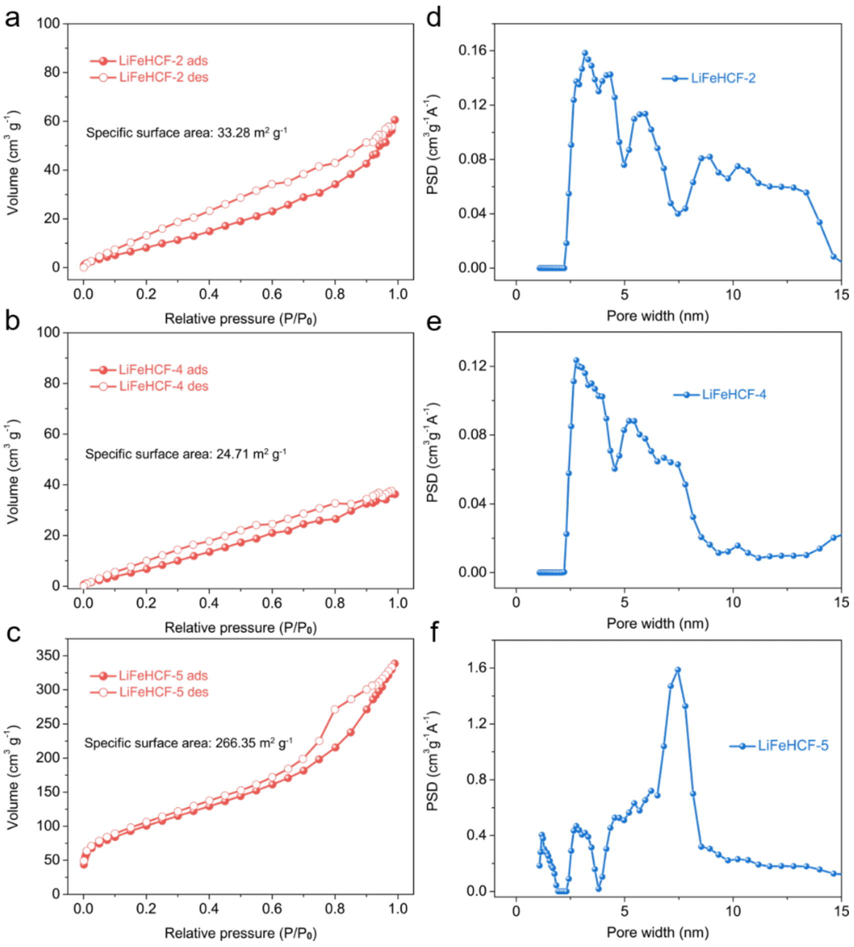

Supplementary Figure 10. Nitrogen adsorption-desorption isotherms and pore size distribution curves of LiFeHCF-2, LiFeHCF-4 and LiFeHCF-5 powder samples.

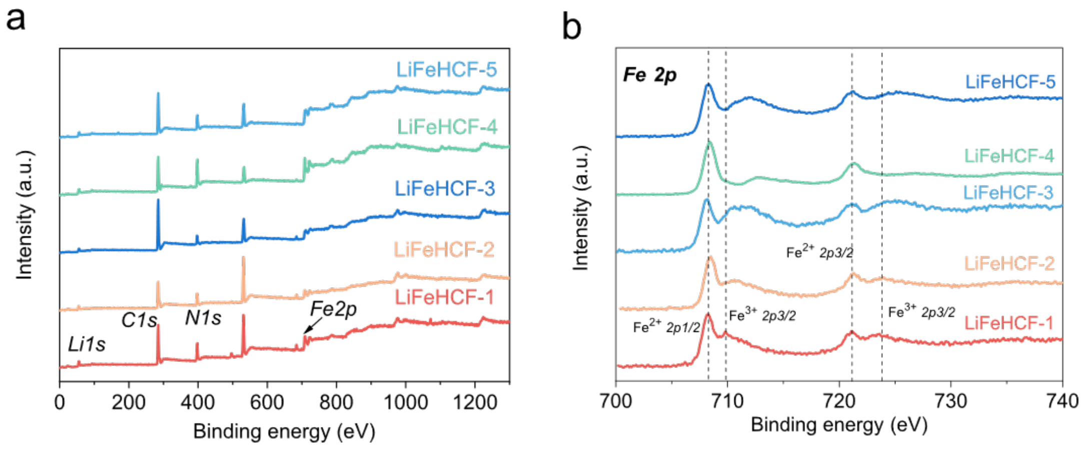

Supplementary Figure 11. Structural characterizations of different LiFeHCF powder samples. (a) XPS survey spectra and (b) XPS spectra of the characteristic Fe 2P peaks.

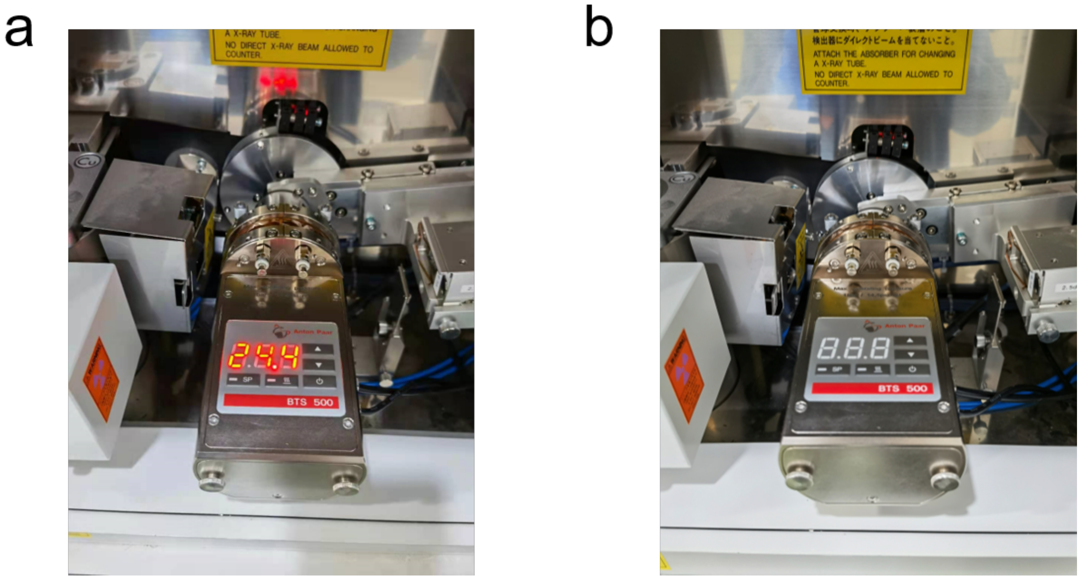

Supplementary Figure 12. Photographic pictures of the X-ray diffraction (XRD) device used for the variable temperature operando measurements.

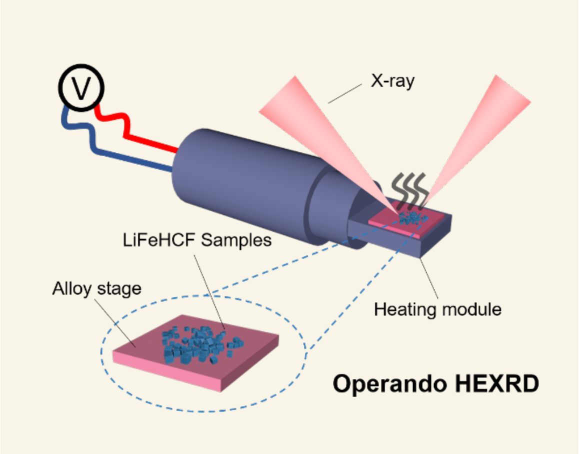

Supplementary Figure 13. Schematic illustration of the variable temperature XRD system used for the operando measurements, the LiFeHCF samples measured was powders.

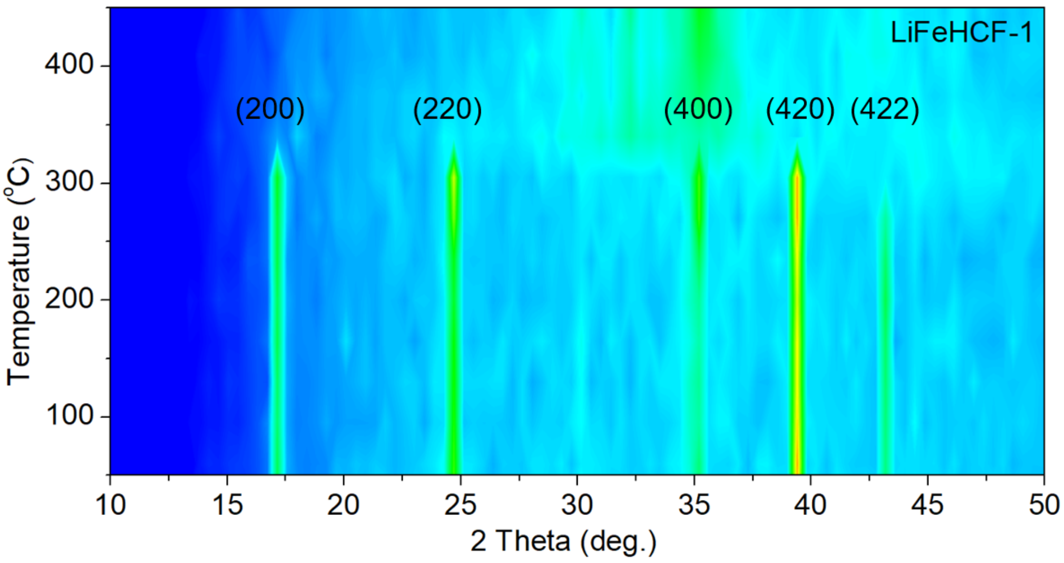

Supplementary Figure 14. Operando phase transformation of the LiFeHCF-1 powder samples. The contour plots of the (200), (220), (400), (420) and (422) reflections during heating from 25 °C to 450 °C.

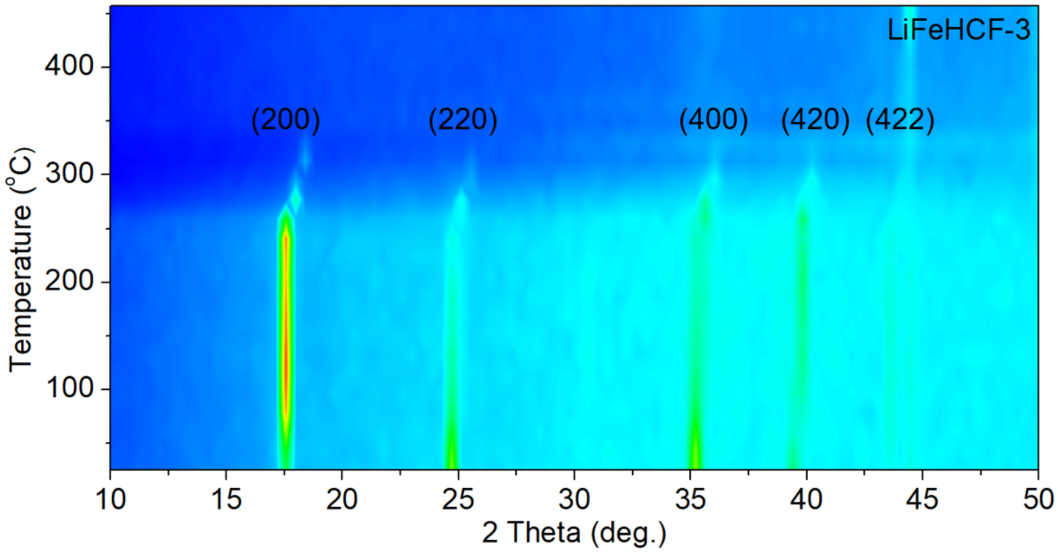

Supplementary Figure 15. Operando phase transformation of the LiFeHCF-3 powder samples. The contour plots of the (200), (220), (400), (420) and (422) reflections during heating from 25 ^{o}C to 450 ^{o}C.

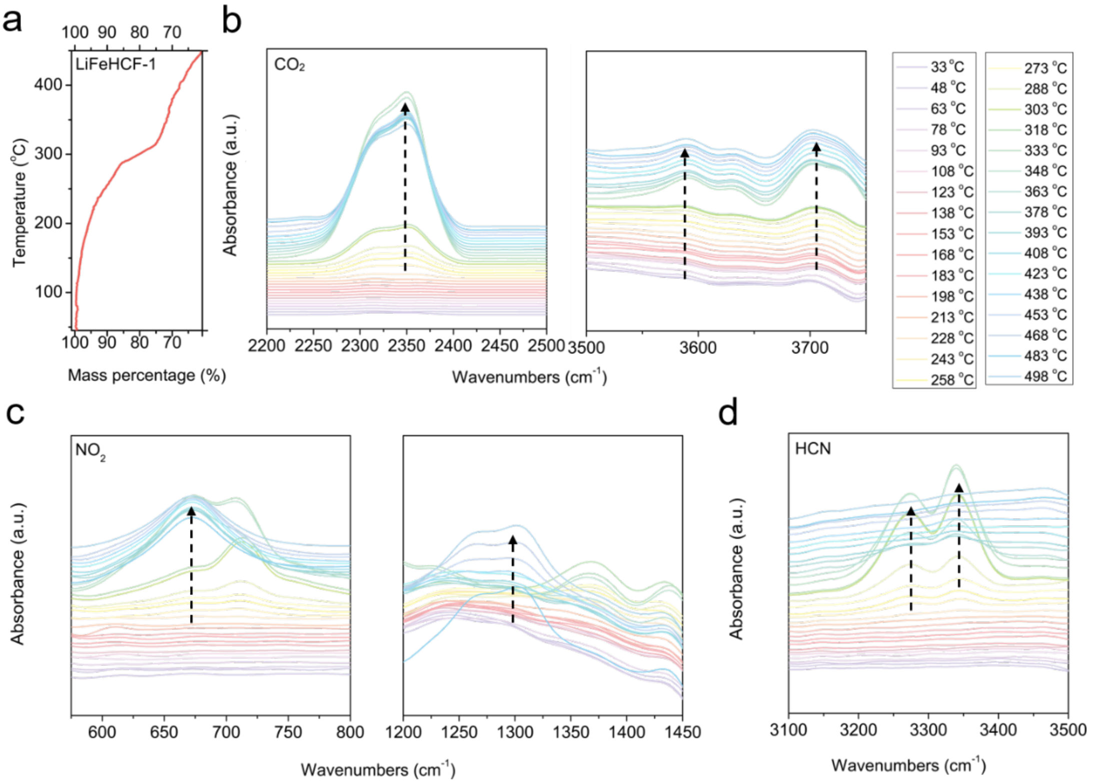

Supplementary Figure 16. Operando combined Thermogravimetry-infrared spectrometry (TGA-IR) analysis: (a) TGA curves of LiFeHCF-1 powder samples. The signal for decomposition products of (b) CO_2, (c) NO_2, (d) HCN during heating from 25 °C to 450 °C.

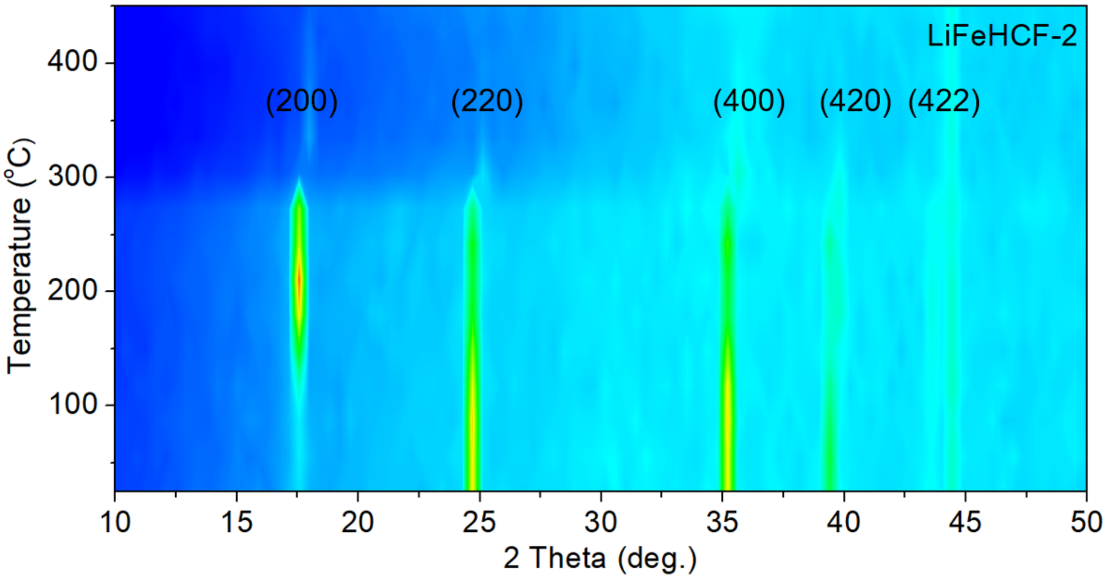

Supplementary Figure 17. Operando phase transformation of the LiFeHCF-2 powder sample. The contour plots of the (200), (220), (400), (420) and (422) reflections from 25 to 450 ^{o}C.

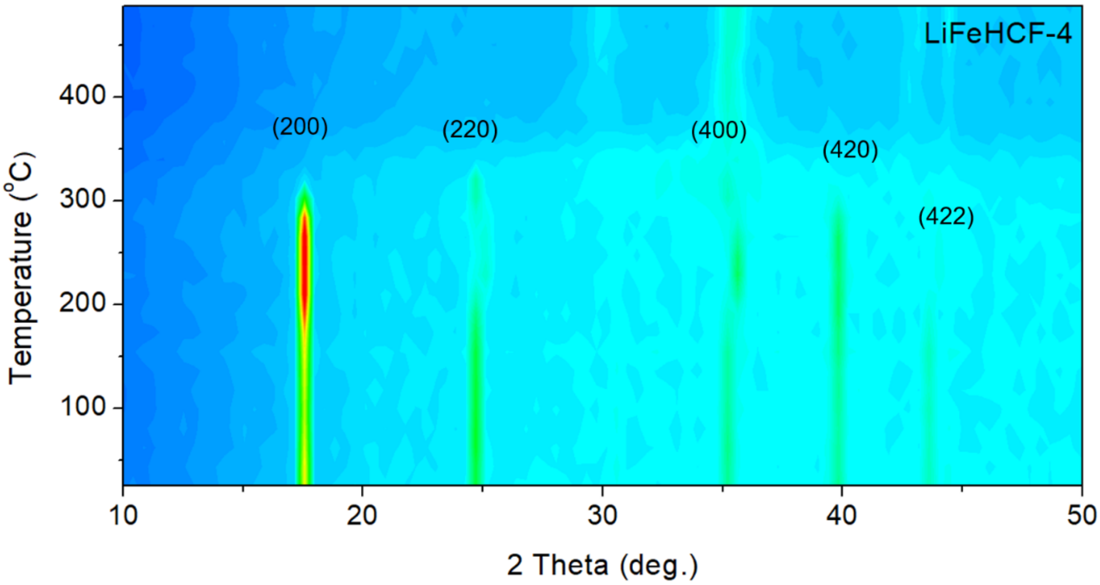

Supplementary Figure 18. Operando phase transformation of the LiFeHCF-4 powder sample. The contour plots of the (200), (220), (400), (420) and (422) reflections from 25 to 450 °C.

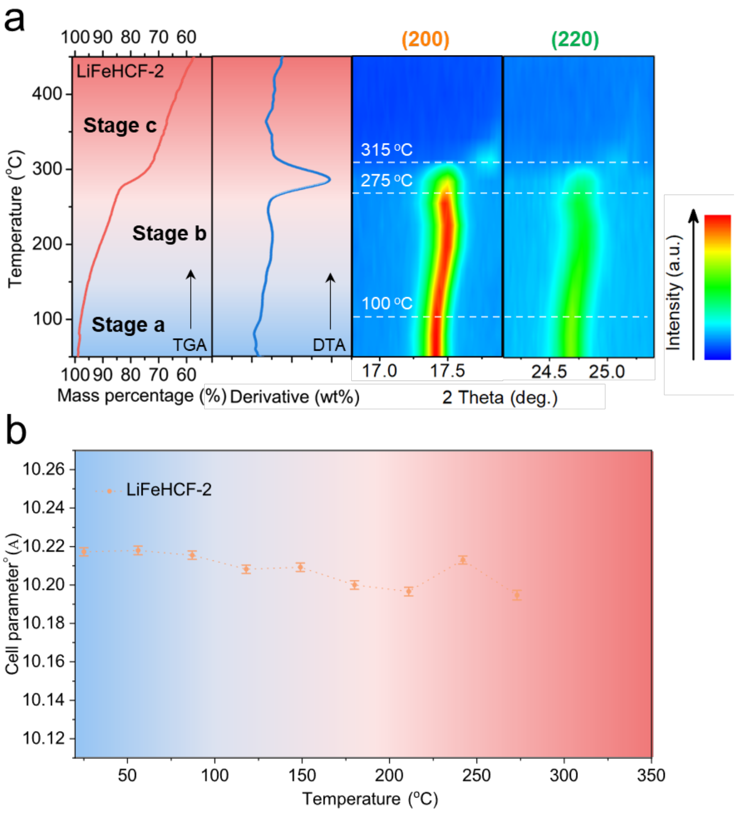

Supplementary Figure 19. (a) Operando monitoring of the structural evolution of LiFeHCF-2 powder from 25 to 450 °C. (b) The cell parameters of distorted frameworks during the heat treatment, the error bars represent the range of cell parameters for the Prussian blue samples upon heat treatment.

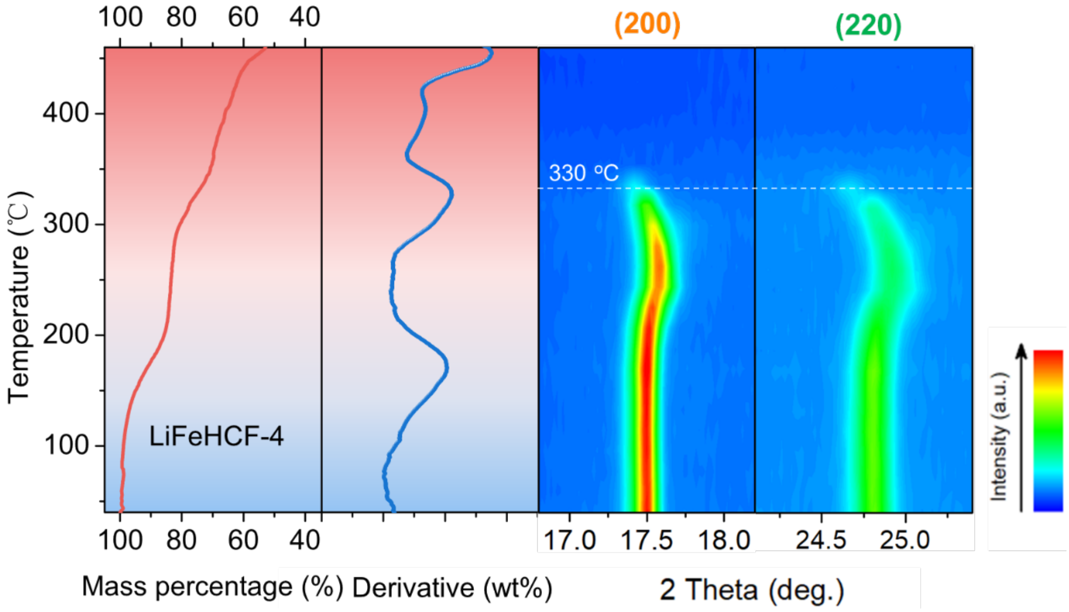

Supplementary Figure 20. Operando monitoring of the structural evolution of LiFeHCF-4 powder from 25 to 450 °C.

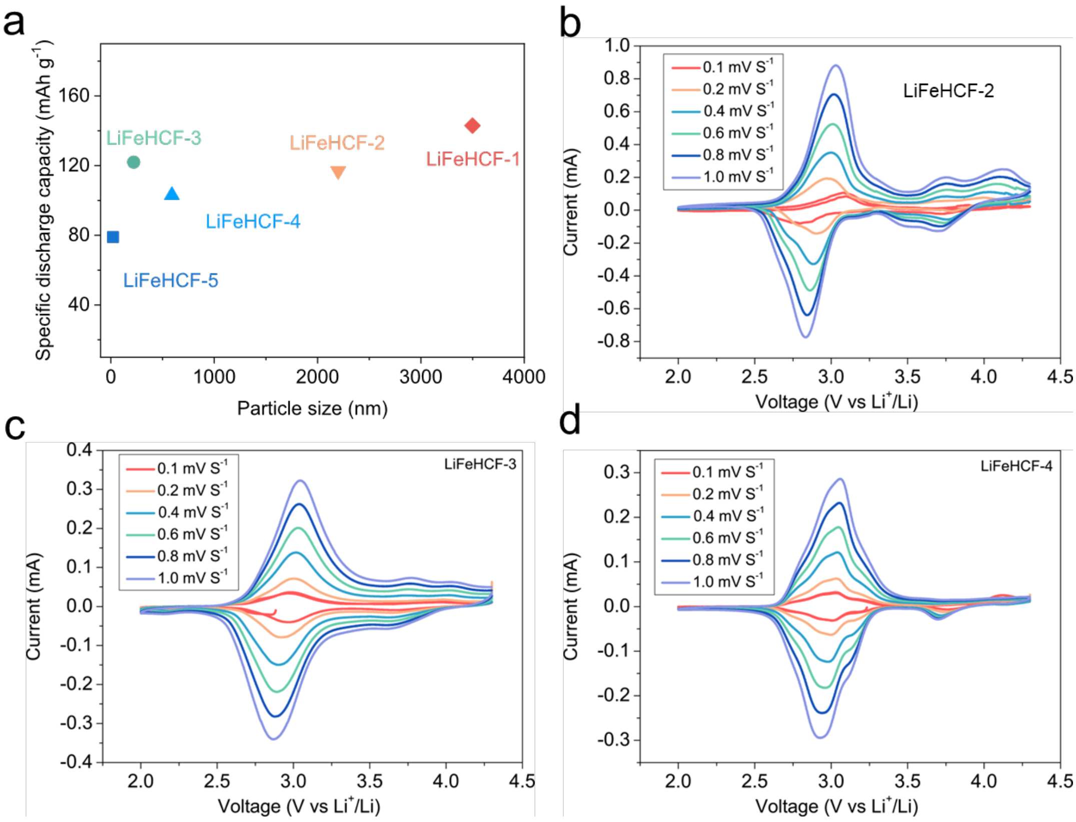

Supplementary Figure 21. (a) Capacity-particle size correlations of LiFeHCF samples (Specific capacity was obtained based on the mass of positive active material). All active material coupled with a Li metal electrode and a $LiPF_6$-containing organic-based electrolyte in coin cell configuration and tested at a specific current of 19 mA g^{-1} at 25 ºC. CV curves of (b) LiFeHCF-2, (c) LiFeHCF-3 and (d) LiFeHCF-4, which tested in CR2032 coin cells at 25 ºC, using lithium metal as the counter electrode.

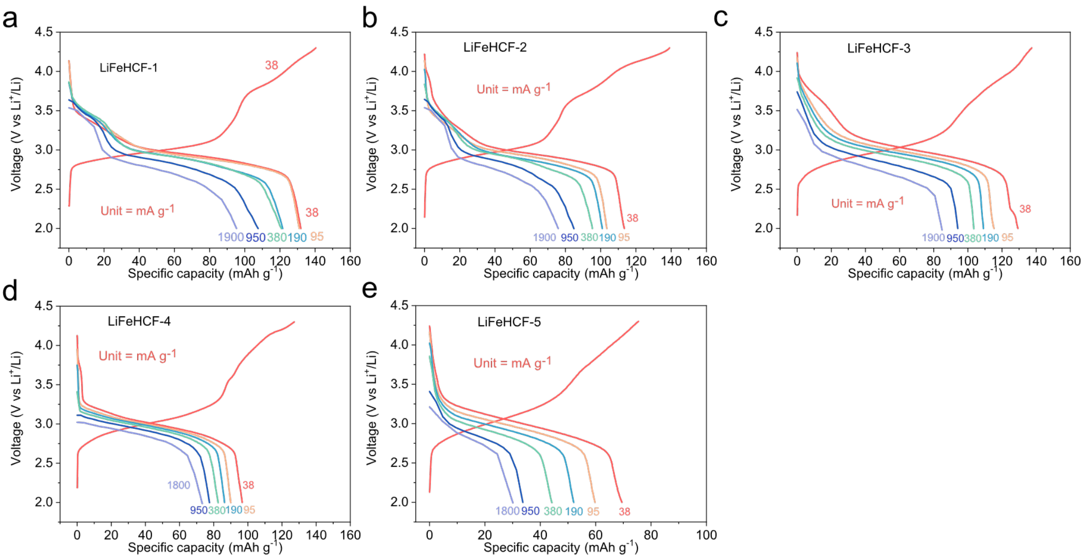

Supplementary Figure 22. Rate performance of LiFeHCF electrodes at various specific currents between 38 and 1900 mA g^{-1}: (a) LiFeHCF-1, (b) LiFeHCF-2, (c) LiFeHCF-3, (d) LiFeHCF-4 and (e) LiFeHCF-5. All samples were tested in CR2032 coin cells at 25 °C and lithium metal was used as the counter electrode (Specific capacity was calculated based on the mass of positive active material).

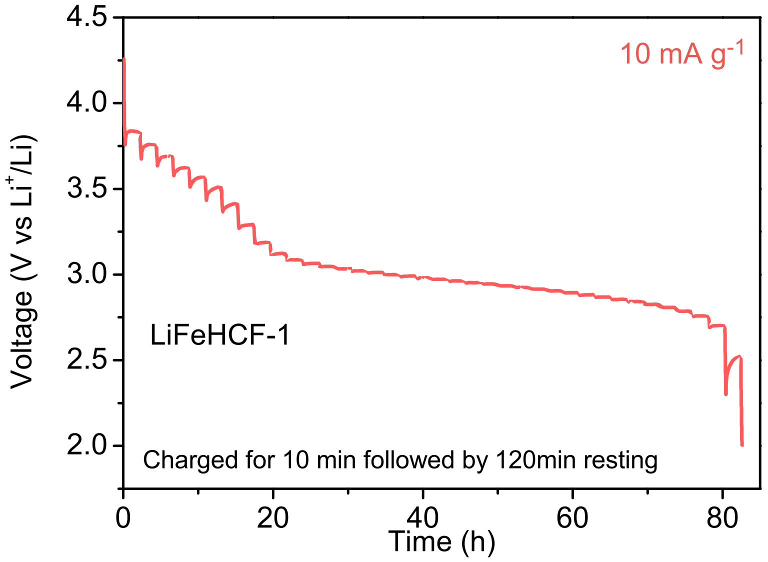

Supplementary Figure 23. GITT curves of the LiFeHCF-1 electrode at the initial discharge process at 10 mA g^{-1}, which tested in CR2032 coin cells at 25 ^{o}C and lithium metal was used as the counter electrode.

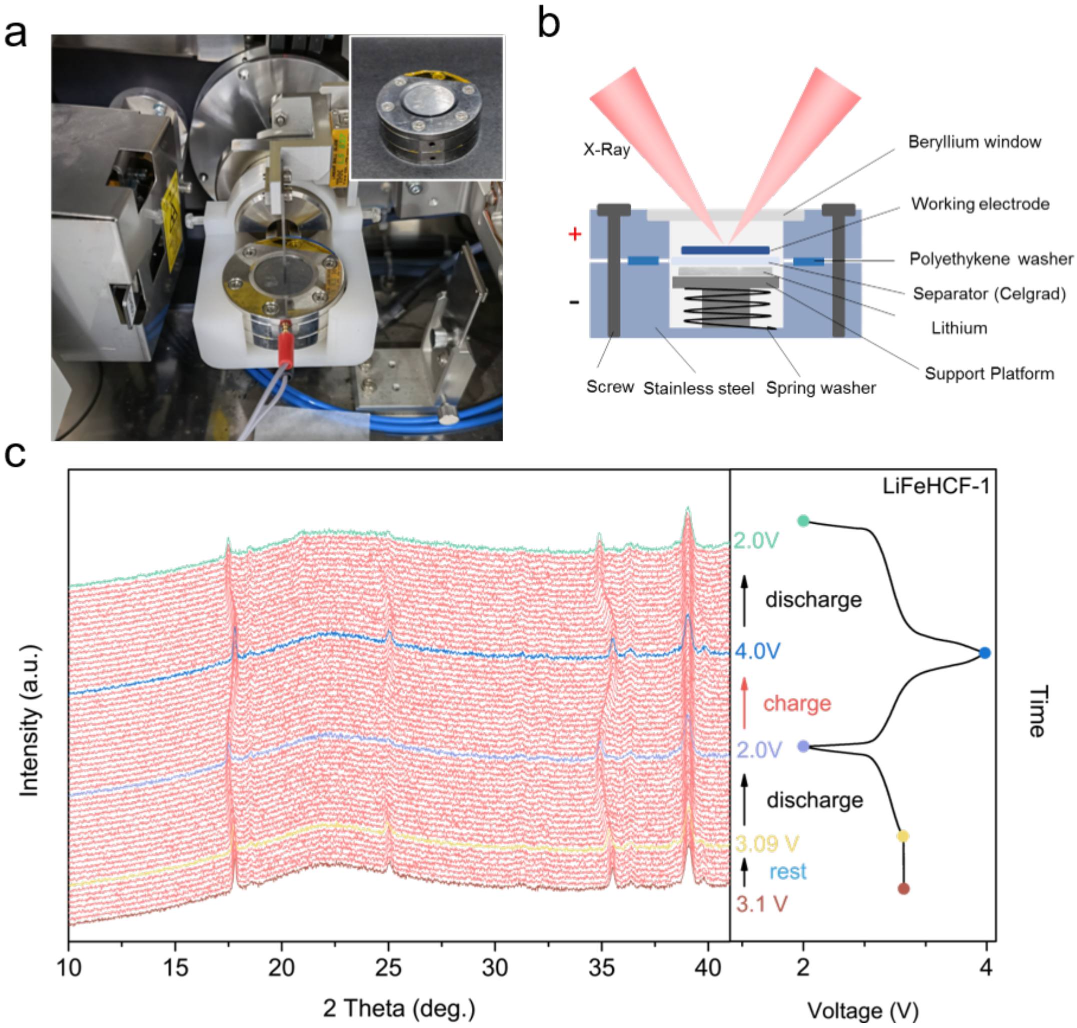

Supplementary Figure 24. (a) The digital image and (b) schematic illustrations of the electrochemical energy storage cell used for the operando XRD measurements. (c) Phase evolution of the LiFeHCF-1 electrode during cycling, the operando battery for XRD testing was carried out by stainless steel cell with beryllium window at 9.5 mA g^{-1} at 25 ^{o}C.

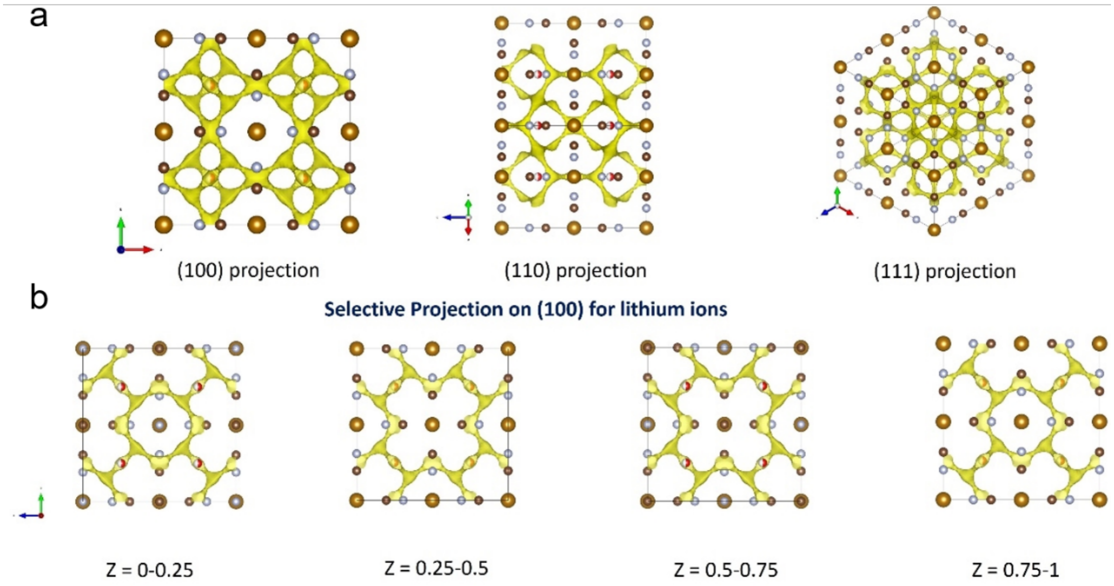

Supplementary Figure 25. Effect of H_2O located at the 8c Wyckoff position of (0.25, 0.25, 0.25) on the Li-ion conduction pathway. (a) Bond Valence Energy 450 Landscape (BVEL) calculation and (b) BVEL with different Z values on the (100) planes for Li-ions in LiFeHCF samples.

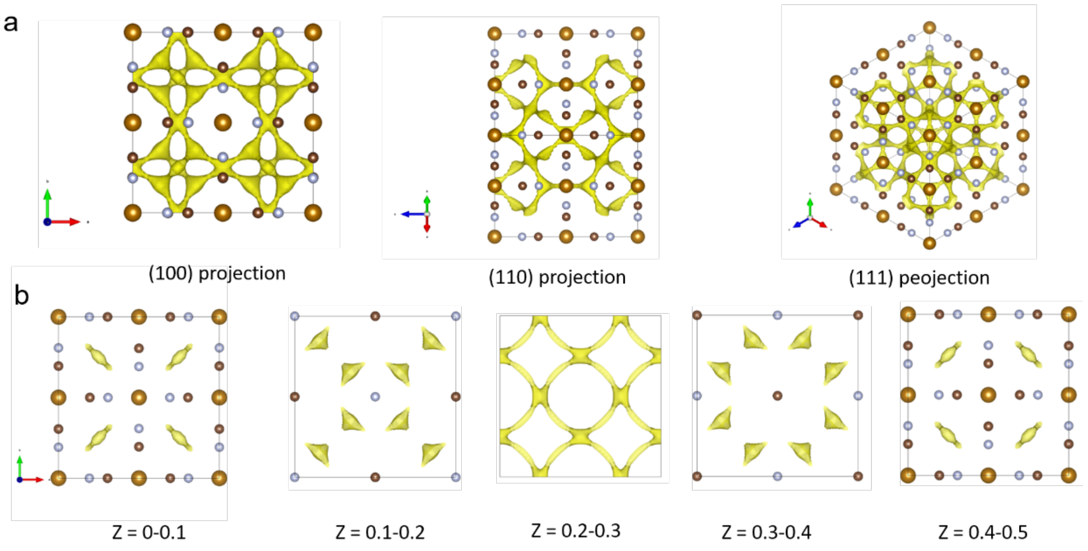

Supplementary Figure 26. (a) Bond Valence Energy 450 Landscape (BVEL) calculation and (b) BVEL with different Z values on the (100) planes for Li-ions in LiFeHCF samples without water molecules.

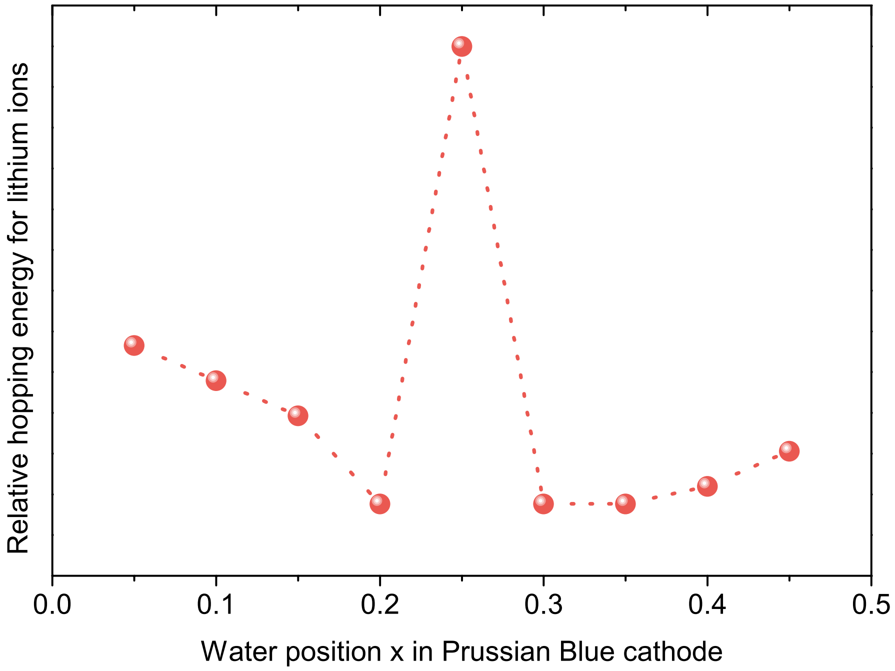

Supplementary Figure 27. Relative hopping energy in different water molecule positions for lithium ion percolation networks.

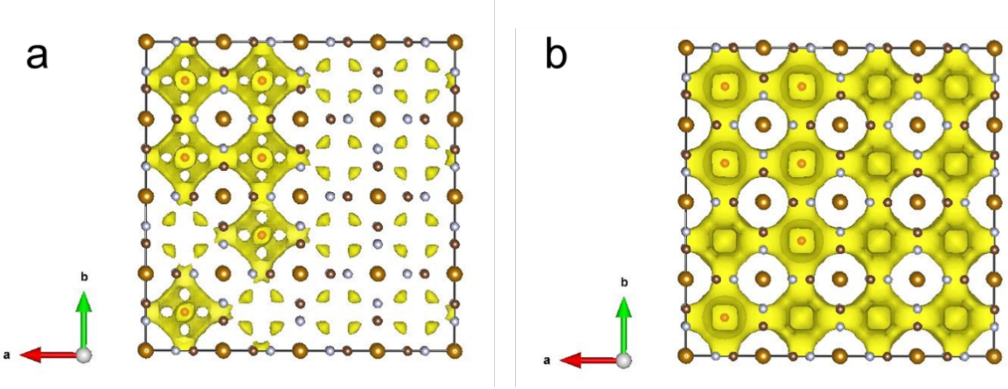

Supplementary Figure 28. Bond valence energy landscapes of a supercell with small mismatch (a) and large mismatch (b).

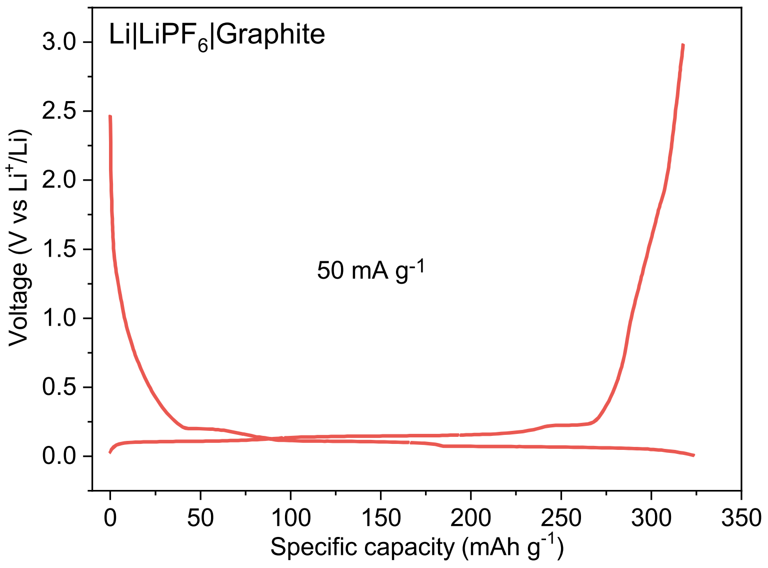

Supplementary Figure 29. Charge and discharge curve of graphite electrode. The graphite was evaluated in coin cell configuration and tested at 25 °C. The specific capacity values were calculated by the mass of negative electrode active material.

Supplementary Table 1. ICP results of LiFeHCF-1, LiFeHCF-2, LiFeHCF-3, LiFeHCF-4, and LiFeHCF-5 samples.

Sample		Li	C	N	H_2O	Chemical formula
1	LiFeHCF-1	2.13 %	21.003%	24.574%	13.4 %	$Li_{1.05}Fe[Fe(CN)_6]_{0.97}·2.6H_2O$
2	LiFeHCF-2	2.6%	20.213%	22.596%	14.6 %	$Li_{1.36}Fe[Fe(CN)_6]_{0.96}·2.9H_2O$
3	LiFeHCF-3	2.34 %	21.323 %	22.589%	25 %	$Li_{1.14}Fe[Fe(CN)_6]_{0.91}·4.7H_2O$
4	LiFeHCF-4	1.33%	20.483 %	24.598%	14.7 %	$Li_{0.63}Fe[Fe(CN)_6]_{0.96}·2.7H_2O$
5	LiFeHCF-5	2.23%	17.276%	18.265%	28%	$Li_{1.0}Fe[Fe(CN)_6]_{0.90}·4.9H_2O$

Supplementary Table 2. Detailed neutron diffraction patterns refinement results of the LiFeHCF-1 powder sample.

$Li_{1.05}Fe[Fe(CN)_6]_{0.97}$•$2.6H_2O$ (LiFeHCF-1)			
symmetry: cubic<br>S.G.: *Fm-3m*<br>a = b = c = 10.2296(2) Å<br>V = 1070.4736 $Å^3$<br>wRp = 2.62%			
Atom	Position	Occupancy	Uiso x 100
Fe	0, 0, 0	0.9863(7)	0.904(9)
Fe	0.5, 0.5, 0.5	0.9863(7)	0.904(9)
C	0.3130(3), 0, 0	0.9702(6)	2.029(8)
N	0.2000(2), 0, 0	0.9702(6)	2.029(8)
O	0.2000(2), 0, 0	0.0298(6)	2.029(8)
O	0.384(3), 0.384(3), 0.384(2)	0.3252(7)	10 (3)
Li	0.25, 0.25, 0.25	0.4362(7)	10 (3)

Supplementary Table 3. Detailed neutron diffraction patterns refinement results of the LiFeHCF-2 powder sample.

$Li_{1.36}Fe[Fe(CN)_6]_{0.96}$•$2.9H_2O$ (LiFeHCF-2)			
symmetry: cubic<br>S.G.: *Fm-3m*<br>a = b = c = 10.2433 Å<br>V = 1074.7645 Å^3<br>wRp = 1.755%			
**Atom**	**Position**	**Occupancy**	**Uiso x 100**
Fe	0, 0, 0	0.98	0.5
Fe	0.5, 0.5, 0.5	0.98	0.5
C	0.3130, 0, 0	0.96	2.0
N	0.1959, 0, 0	0.96	2.0
O	0.1965, 0, 0	0.096	2.0
O	0.373, 0.373, 0.373	0.1512	6.9
Li	0.25, 0.25, 0.25	0.34	8

Supplementary Table 4. Detailed neutron diffraction patterns refinement results of the LiFeHCF-3 powder sample.

$Li_{1.14}Fe[Fe(CN)_6]_{0.91}$•4.7$H_2O$ (LiFeHCF-3)			
symmetry: cubic<br>S.G.: *Fm-3m*<br>a = b = c = 10.2134(2) Å<br>*V* = 1065.40(2) $Å^3$<br>wRp = 2.35%			
Atom	Position	Occupancy	Uiso x 100
Fe	0, 0, 0	0.9666(7)	1.839(9)
Fe	0.5, 0.5, 0.5	0.9666(7)	1.839(9)
C	0.3130(3), 0, 0	0.9119(6)	3.244(8)
N	0.1984(2), 0, 0	0.9119(6)	3.244(8)
O	0.1984(2), 0, 0	0.0881(6)	3.244(8)
O	0.366(3), 0.366(3),0.366(2)	0.5228(7)	10 (3)
Li	0.25, 0.25, 0.25	0.5758(7)	10 (3)

Supplementary Table 5. Detailed neutron diffraction patterns refinement results of the LiFeHCF-4 powder sample.

$Li_{0.63}Fe[Fe(CN)_6]_{0.96}$•$2.7H_2O$ (LiFeHCF-4)			
symmetry: cubic<br>S.G.: *Fm-3m*<br>a = b = c = 10.1818(4) Å<br>*V* = 1055.54(4) Å^3<br>wRp = 3.23%			
Atom	Position	Occupancy	Uiso x 100
Fe	0, 0, 0	1	1.074(9)
Fe	0.5, 0.5, 0.5	1	1.074(9)
C	0.3113(3), 0, 0	0.9633	3.360(8)
N	0.1965(4), 0, 0	0.9633	3.360(8)
O	0.1965(4), 0, 0	0.0367	3.360(8)
O	0.366(3), 0.366(3), 0.366(2)	0.3125	8 (4)
Li	0.25, 0.25, 0.25	0.3250	8 (4)

Supplementary Table 6. Comparison of the synthetic method as well as electrochemical performances of PBA cathodes in literatures.

Positive electrode	Synthetic method	Cell configuration	Negative electrode	Electrolyte	Initial specific capacity	Cycling performance	Ref
NaFeHCF (70 wt% in electrode, 2 mg cm^{-2})	Modified Coprecipitation	Coin cell (type 2032)	Sodium metal	1 M $NaClO_4$ (EC:PC, 1:1 by volume with 3 % FEC)	116 mAh g^{-1} (10 mA g^{-1})	71 mAh·g^{-1} (0.1 A g^{-1}) 71% after 500 cycles	[6]
NaFeMnNiCuCoHCF (70 wt% in electrode, 2.0-2.5 mg cm^{-2})	Coprecipitation	Coin cell (type 2032)	Sodium metal	1 M $NaClO_4$ (EC:DMC:PC, 1:1:1 by volume with 5% FEC)	120 mAh g^{-1} (10 mA g^{-1})	70 mAh·g^{-1} (0.5 A g^{-1}) after 1000 cycles	[7]
EDTA-NaMnFeHCF (70 wt% in electrode, 2 mg cm^{-2})	Modified Coprecipitation	Coin cell (type 2032)	Sodium metal	1 M $NaPF_6$ (EC:PC, 1:1 by volume with 2% FEC)	137 mAh g^{-1} (25 mA g^{-1})	~80 mAh·g^{-1} (0.5 A g^{-1}) 80.9% after 1000 cycles	[8]
NaNiFeHCF (70 wt% in electrode, 4 mg cm^{-2})	Modified Coprecipitation	Coin cell (type 2032)	Sodium metal	1 M $NaClO_4$ (EC:DEC, 1:1 by volume with 5% FEC)	83 mAh g^{-1} (8.5 mA g^{-1})	~58 mAh·g^{-1} (0.8 A g^{-1}) after 1000 cycles	[9]
NaFeHCF (70 wt% in electrode, 2 mg cm^{-2})	Hydrothermal Method	Coin cell (type 2032)	Sodium metal	1 M $NaClO_4$ (EC:DEC, 1:1 by volume with 2% FEC)	82.5 mAh g^{-1} (50 mA g^{-1})	~65 mAh·g^{-1} (0.5 A g^{-1}) 71.1 % after 1000 cycles	[10]
NaFeHCF@C (90 wt% in electrode, 1-2 mg cm^{-2})	Self-oxidation and Carbon Coating	Coin cell (type 2025)	Sodium metal	1 M $NaPF_6$ (EC:DEC, 1:1 by volume)	130 mAh g^{-1} (50 mA g^{-1})	90 mAh·g^{-1} (2.0 A g^{-1}) 90% after 2000 cycles	[11]
HT-NaFeHCF (70 wt% in electrode, 2 mg cm^{-2})	Coprecipitation and Heat-treatment	Coin cell (type 2032)	Sodium metal	1 M $NaClO_4$ (EC:PC, 1:1 by volume with 3 % FEC)	85 mAh g^{-1} (50 mA g^{-1})	~80 mAh·g^{-1} (0.5 A g^{-1}) after 1000 cycles	[12]
**LiFeHCF (70 wt% in electrode, 1.5 mg cm^{-2})**	**Ion-exchange Method**	**Coin cell (type 2032)**	**Lithium metal**	**1 M $LiPF_6$ (EC:DEC, 1:1 by volume)**	**143 mAh g^{-1} (19 mA g^{-1})**	**95 mAh·g^{-1} (1.9 A g^{-1}) 80.7% after 1000 cycles**	**This work**

Supplementary Table 7 Summary of the effect of water molecule position on the lithium-ion percolation networks.

	Projection on (100)	Projection on (110)	Projection on (111)
H_2O at (0.05, 0.05, 0.05)	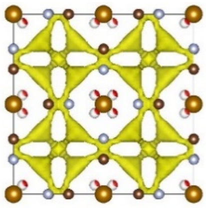	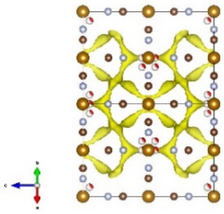	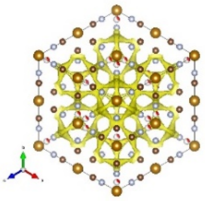
H_2O at (0.1, 0.1, 0.1)	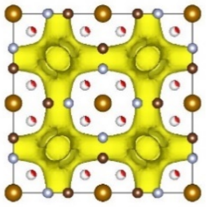	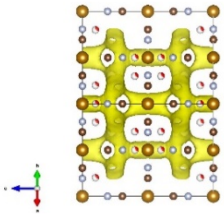	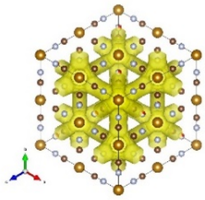
H_2O at (0.15, 0.15, 0.15)	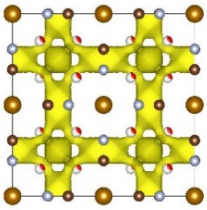	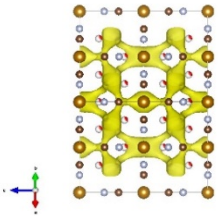	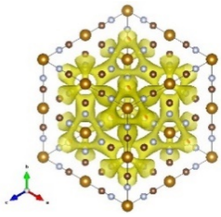
H_2O at (0.2, 0.2, 0.2)	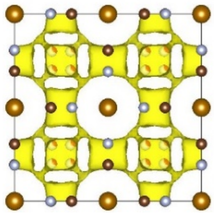	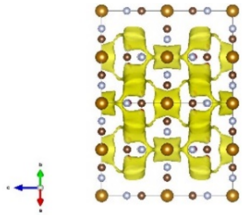	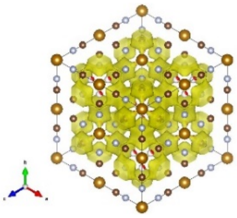
H_2O at (0.25, 0.25, 0.25)	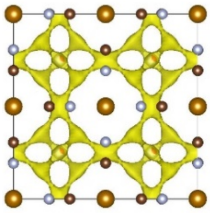	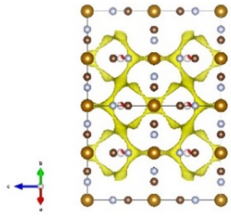	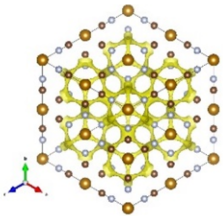
H_2O at (0.3, 0.3, 0.3)	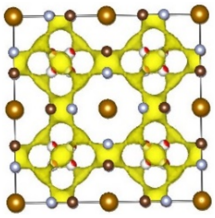	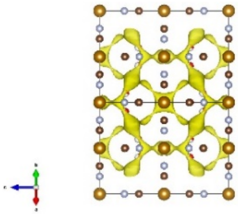	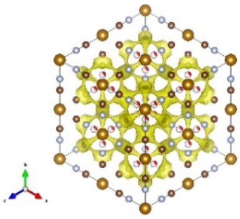

H_2O at (0.35, 0.35, 0.35)	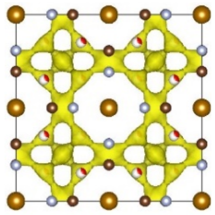	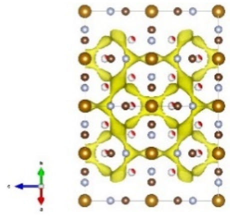	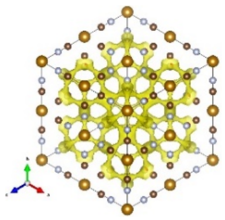
H_2O at (0.4, 0.4, 0.4)	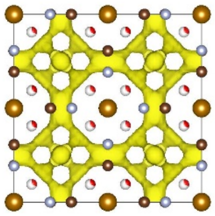	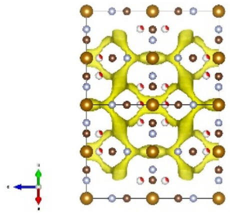	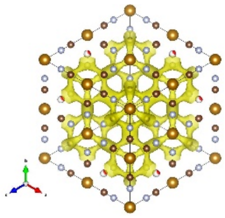
H_2O at (0.45, 0.45, 0.45)	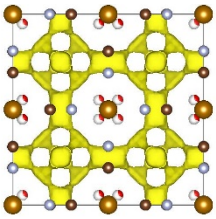	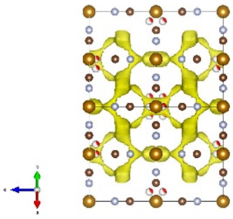	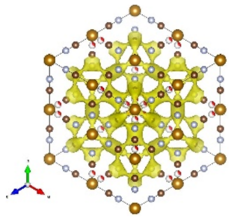

References

[1] Mizushima, K., Jones, P. C., Wiseman, P. J. & Goodenough, J. B. Li_xCoO_2 (0 < x ⩽ 1): A new cathode material for batteries of high energy density. *Solid State Ionics* **3** 171-174 (1981).

[2] Liu, Z. L., Yu, A., & Lee, J. Y. Synthesis and characterization of $LiNi_{1-x-y}Co_xMn_yO_2$ as the cathode materials of secondary lithium batteries. *J. Power Sources* **81** 416-419 (1999).

[3] Padhi, A. K., Nanjundaswamy, K. S. & Goodenough, J. B. Phospho-olivines as positive-electrode materials for rechargeable lithium batteries. *J. Electrochem. Soc.* **144**, 1188-1194 (1997).

[4] Liu, Y. et al. Sodium storage in Na-rich $Na_xFeFe(CN)_6$ nanocubes. *Nano Energy* **12**, 386-393 (2015).

[5] He, G. & Nazar, L. F. Crystallite size control of Prussian white analogues for nonaqueous potassium-ion batteries. *ACS Energy Lett.* **2**, 1122-1127 (2017).

[6] Wang, W. *et al.* Reversible structural evolution of sodium-rich rhombohedral Prussian blue for sodium-ion batteries. *Nat. Commun.* **11**, 980 (2020).

[7] Ma, Y. *et al.* High-entropy metal–organic frameworks for highly reversible sodium storage. *Adv. Mater.* **33**, 2101342 (2021).

[8] Shang, Y. *et al.* Unconventional Mn vacancies in Mn–Fe Prussian blue analogs: suppressing Jahn-Teller distortion for ultrastable sodium storage. *Chem* **6**, 1804-1818 (2020).

[9] Peng, J. *et al.* Defect-free-induced Na^+ disordering in electrode materials. *Energy Environ. Sci.* **14**, 3130-3140 (2021).

[10] Sun, J. *et al.* Elevating the discharge plateau of prussian blue analogs through low-spin Fe redox induced intercalation pseudocapacitance. *Energy Storage Mater.* **43**, 182-189 (2021).

[11] Jiang, Y. *et al.* Prussian Blue@C composite as an ultrahigh-rate and long-life sodium-ion battery cathode. *Adv. Funct. Mater.* **26**, 5315-5321 (2016).

[12] Wang, W. *et al.* Effect of eliminating water in Prussian blue cathode for sodium-

ion batteries. *Adv. Funct. Mater.* https://doi.org/10.1002/adfm.2021117 (2022).
